# Abscisic acid promotes auxin biosynthesis to inhibit primary root elongation in rice

**DOI:** 10.1093/plphys/kiac586

**Published:** 2022-12-19

**Authors:** Hua Qin, Juan Wang, Jiahao Zhou, Jinzhu Qiao, Yuxiang Li, Ruidang Quan, Rongfeng Huang

**Affiliations:** Biotechnology Research Institute, Chinese Academy of Agricultural Sciences, Beijing 100081, China; National Key Facility of Crop Gene Resources and Genetic Improvement, Beijing 100081, China; Biotechnology Research Institute, Chinese Academy of Agricultural Sciences, Beijing 100081, China; National Key Facility of Crop Gene Resources and Genetic Improvement, Beijing 100081, China; Biotechnology Research Institute, Chinese Academy of Agricultural Sciences, Beijing 100081, China; Biotechnology Research Institute, Chinese Academy of Agricultural Sciences, Beijing 100081, China; Biotechnology Research Institute, Chinese Academy of Agricultural Sciences, Beijing 100081, China; Biotechnology Research Institute, Chinese Academy of Agricultural Sciences, Beijing 100081, China; National Key Facility of Crop Gene Resources and Genetic Improvement, Beijing 100081, China; Biotechnology Research Institute, Chinese Academy of Agricultural Sciences, Beijing 100081, China; National Key Facility of Crop Gene Resources and Genetic Improvement, Beijing 100081, China

## Abstract

Soil compaction is a global problem causing inadequate rooting and poor yield in crops. Accumulating evidence indicates that phytohormones coordinately regulate root growth via regulating specific growth processes in distinct tissues. However, how abscisic acid (ABA) signaling translates into auxin production to control root growth during adaptation to different soil environments is still unclear. In this study, we report that ABA has biphasic effects on primary root growth in rice (*Oryza sativa*) through an auxin biosynthesis-mediated process, causing suppression of root elongation and promotion of root swelling in response to soil compaction. We found that ABA treatment induced the expression of auxin biosynthesis genes and auxin accumulation in roots. Conversely, blocking auxin biosynthesis reduced ABA sensitivity in roots, showing longer and thinner primary roots with larger root meristem size and smaller root diameter. Further investigation revealed that the transcription factor basic region and leucine zipper 46 (OsbZIP46), involved in ABA signaling, can directly bind to the *YUCCA8/rice ethylene-insensitive 7 (OsYUC8/REIN7)* promoter to activate its expression, and genetic analysis revealed that *OsYUC8/REIN7* is located downstream of *OsbZIP46*. Moreover, roots of mutants defective in ABA or auxin biosynthesis displayed the enhanced ability to penetrate compacted soil. Thus, our results disclose the mechanism in which ABA employs auxin as a downstream signal to modify root elongation and radial expansion, resulting in short and swollen roots impaired in their ability to penetrate compacted soil. These findings provide avenues for breeders to select crops resilient to soil compaction.

## Teaser

Abscisic acid activates auxin biosynthesis to inhibit root elongation and promote root swelling in response to soil compaction.

## Introduction

The root system, a crucial belowground plant organ, mediates water and nutrient uptake and provides mechanical support for shoot growth (de Dorlodot et al., 2007). An extensively developed root system improves the ability of a plant to obtain nutrients and resist environmental stresses, and therefore can affect crop productivity (Uga et al., 2013; Shekhar et al., 2019; Kitomi et al., 2020). Root growth is affected by various factors, one of which is soil compaction, a major constraint for soil exploration and resource capture by plants roots (Lipiec et al., 2012; Correa et al., 2019). Soil compaction reduces crop yield by ∼25% and plants with roots that are able to penetrate hard soil have an advantage in water and nutrient capture at depth, ultimately affording superior performance under drought or low soil fertility (Barken et al., 1987; Uga et al., 2013; Lynch, 2018). Therefore, breeding crops to better withstand compacted soil offers a genetic solution to improve root growth during mechanical impedance (Schneider et al., 2021). Recent studies show that soil compaction inhibits root elongation and promotes radical expansion of roots by restricting ethylene diffusion (Pandey et al., 2021), and several hormone signals including auxin and abscisic acid (ABA) have been reported to function downstream of ethylene to inhibit root elongation (Ma et al., 2014; Yin et al., 2015; Qin et al., 2017; Huang et al., 2022), suggesting that ethylene may direct these hormones to modulate root growth in compacted soil.

ABA is a major abiotic stress-responsive hormone and plays an essential role in root growth in plants (Wang et al., 2017; Takatsuka and Umeda, 2019). Works in Arabidopsis (*Arabidopsis thaliana*) have identified a core ABA signaling pathway (Umezawa et al., 2010). The ABA receptors PYRABACTIN RESISTANCE1/PYRABACTIN RESISTANCE1-LIKE/REGULATORY COMPONENT OF ABA RECEPTOR 1 (PYR1/PYL/RCAR1) interact with and inhibit clade-A protein phosphatase type 2Cs (PP2Cs) in the presence of ABA, leading to the activation of SNF1-related type 2 protein kinases (SnRK2s), which further phosphorylate and activate basic region and leucine zipper (bZIP) transcription factors to regulate the expression of ABA-responsive genes (Ma et al., 2009; Park et al., 2009; Umezawa et al., 2009; Cutler et al., 2010). To date, both positive and negative effects of ABA on root growth have been documented, depending on root types and ABA concentrations (Luo et al., 2014; Wang et al., 2017). Typically, low concentrations of ABA stimulate but high concentrations inhibit root elongation (Li et al., 2017).

Auxin is another vital hormone and is generally recognized as a master regulator in plant root development (Saini et al., 2013; Olatunji et al., 2017; Lv et al., 2021a). Indole-3-acetic acid (IAA) is the predominant form of auxin in plants, and it is synthesized through a simple two-step pathway. Briefly, tryptophan (Trp) is first converted to indole-3-pyruvic acid (IPyA) by the TRYPTOPHAN AMINOTRANSFERASE OF ARABIDOPSIS (TAA) family of aminotransferases, IPyA is then converted into IAA by the YUCCA (YUC) family of flavin monooxygenases (Mashiguchi et al., 2011; Won et al., 2011). In rice (*Oryza sativa*), overexpression or mutation of *TAA1* or *YUC* genes leads to abnormal auxin content and root growth (Woo et al., 2007; Yamamoto et al., 2007; Yoshikawa et al., 2014; Qin et al., 2017; Zhang et al., 2018), indicating that *TAA* and *YUC* gene families play a key role in auxin biosynthesis and root growth in rice.

The crosstalk between ABA and auxin to regulate root growth has been evidenced by many studies (Thole et al., 2014; Li et al., 2017; Wang et al., 2017). ABA treatment induced the expression of auxin biosynthesis genes and *DR5-GUS* in roots (Wang et al., 2017). Mutations in auxin resistant (*AXR*) genes or auxin transport genes resulted in insensitive to ABA on root growth (Wilson et al., 1990; Belin et al., 2009; Liu et al., 2013; Li et al., 2017). Although accumulating evidence began to highlight the interaction between ABA and auxin in root growth, the molecular mechanisms underlying are far from being well understood. In this study, we report that ABA regulates rice root growth in compacted soil via modulating auxin accumulation in roots. OsbZIP46, a downstream bZIP transcription factor in ABA signaling pathway, functions as a crosstalk node between ABA and auxin in root growth through directly binding to the *OsYUC8/REIN7* promoter to activate its expression. Our results reveal the molecular mechanism that ABA regulates auxin biosynthesis to modulate root growth in response to soil compaction, which could provide a pathway for breeders to select crops resilient to soil compaction.

## Results

### ABA inhibits root elongation and promotes root swelling in response to soil compaction

**Figure 1:**
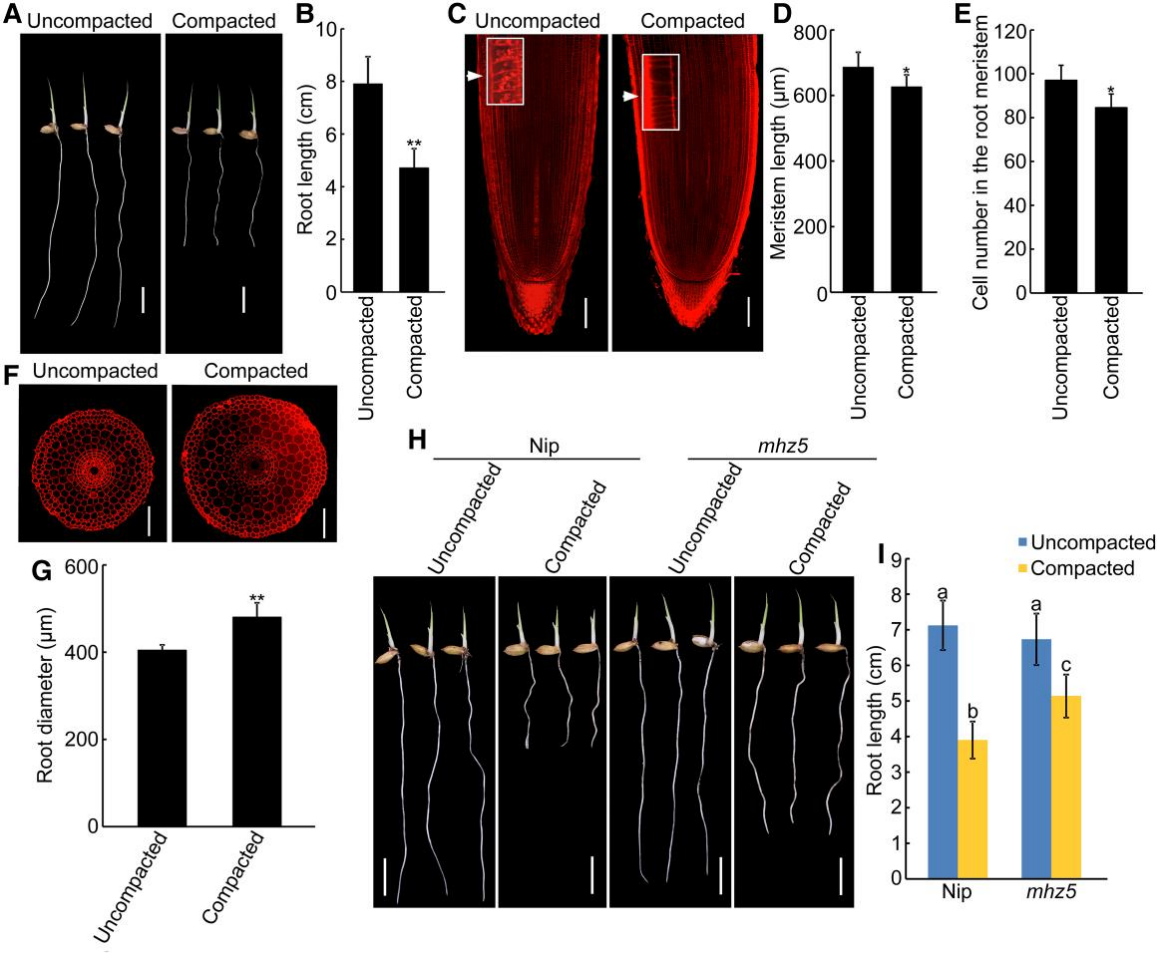
ABA is involved in soil compaction-modulated root growth. (A and B) Morphology (A) and primary root length (B) of 4-d-old wild-type (WT) seedlings grown in uncompacted or compacted soil. Bars = 1 cm. Data are means ± SD (*n* ≥ 30 independent seedlings). The individual images in (A) were digitally extracted for comparison. C, Representative propidium iodide staining of longitudinal sections of root tips of 4-d-old WT seedlings grown in uncompacted or compacted soil. White arrows indicate the proximal end of the root meristem. White rectangle insets are an enlargement (three times magnification) of the regions at the proximal end of the root meristem. Bars = 100 μm. (D and E) Length (D) and cortical cell number (E) of the root meristem zones of the corresponding seedlings indicated in panel (C). Data are means ± SD (*n* ≥ 10 independent seedlings). F, Representative propidium iodide staining of radial sections of root elongation zone of 4-d-old WT seedlings grown in uncompacted or compacted soil. Bars = 100 μm. G, Root diameter of the corresponding seedlings indicated in panel (F). Data are means ± SD (*n* ≥ 10 independent seedlings). (H and I) Morphology (H) and primary root length (I) of 4-d-old Nip and *mhz5* seedlings grown in uncompacted or compacted soil. Bars = 1 cm. Data are means ± SD (*n* ≥ 30 independent seedlings). The individual images in (H) were digitally extracted for comparison. Different letters indicate significant differences (*P* < 0.05, one-way ANOVA with Tukey's test). Asterisks in (B), (D), (E) and (G) indicate significant differences compared with uncompacted values at *P < 0.05 and **P < 0.01 (Student's *t*-test).

The findings that soil compaction inhibits root growth through restricting ethylene diffusion (Pandey et al., 2021) and ABA functions downstream of ethylene to inhibit root growth (Ma et al., 2014; Yin et al., 2015) inspired us to investigate whether ABA is involved in regulating root growth in response to soil compaction. To explore this hypothesis, we firstly observed the roots growing in compacted and uncompacted soil. Consistent with previous reports (Pandey et al., 2021), soil compaction significantly inhibits root growth (Figure 1, A and B). To further clarify the cellular basis of the inhibitory effect of soil compaction on root growth, we prepared longitudinal and transversal sections of root tips and maturation zones, and stained these with propidium iodide (PI). Compared with roots growing in uncompacted condition, roots grown in compacted soil exhibited a significant decrease in root meristem size (∼13%), meristem zone cell number (∼15%) and maturation zone cell length (∼21%), and an increase in root diameter (∼19%) (Figure 1, C–G and Supplemental Figure S1, A, B), indicating that soil compaction has biphasic effects on root growth, namely, inhibiting root elongation and promoting root swelling, and the inhibition of soil compaction on root elongation results from reduced cell division in meristem and cell elongation in maturation zones.

Recent evidence indicates that ABA treatment inhibits cell proliferation and promotes radical expansion of cortical cells in the root meristem (Huang et al., 2021), which is similar to the impact of soil compaction, suggesting that ABA may function in soil compaction modulated root growth. Therefore, we further investigated the effect of ABA on root growth. Our results showed that exogenous ABA treatment resulted in a decrease in root length (∼33%), meristem size (∼26%), meristem zone cell number (∼20%) and maturation zone cell length (∼33%), and an increase in root diameter (∼27%) (Supplemental Figures S2, A–G and S3, A and B), which phenocopied the impact of soil compaction. These results indicate that ABA might be involved in root growth under compacted soil conditions. Further investigation of rice ABA biosynthetic mutant *mhz5* showed that the *mhz5* roots inhibition is less than that of the wild type when grown in compacted soil (Figure 1, H and I). These results indicate that ABA is required for root growth in response to soil compaction conditions.

### Disrupting auxin biosynthesis weakens root response to ABA and soil compaction

**Figure 2:**
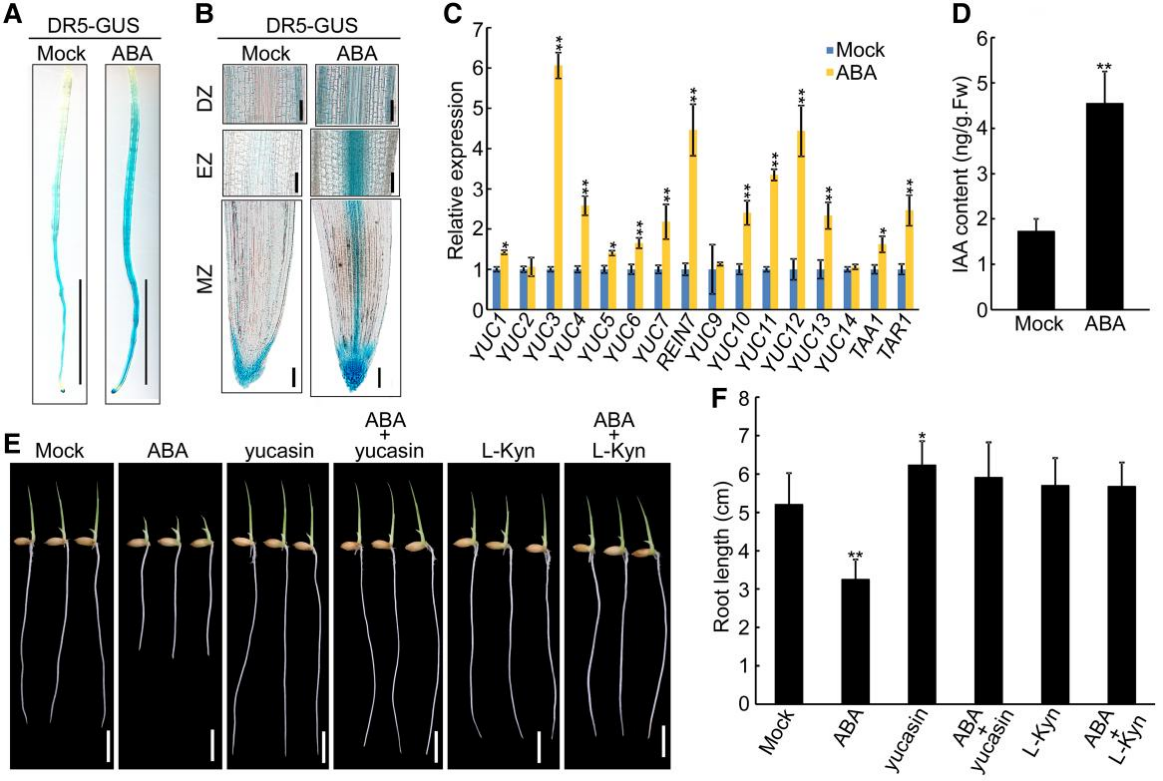
ABA promotes auxin accumulation to inhibit root growth. A, Representative images of *DR5-GUS* expression in root with or without ABA treatment. Seedlings of 4-d-old transgenic lines containing *DR5-GUS* were treated with or without 1 μM ABA for 12 h before GUS activity was assayed. Bar = 1 cm. B, Representative longitudinal sections of meristematic zone (MZ), elongation zone (EZ) and differentiation zone (DZ) in panel (A). Bar = 100 μm. C, Expression of *YUCs*, *TAA1* and *TAR1* in the roots of 4-d-old wild-type seedlings treated with or without 1 μM ABA for 4 h. The data are shown as mean ± SD; *n* = 3 biological replicates. D, Indole-3-acetic acid (IAA) contents in 4-d-old wild-type seedlings treated with or without 1 μM ABA for 24 h. The data are shown as mean ± SD; *n* = 3 biological replicates. (E and F) Morphology (E) and primary root length (F) of 4-d-old wild-type seedlings grown in the absence or presence of 0.5 μM ABA, with or without supplementation of 5 μM yucasin or L-Kyn. Bar = 1 cm. Data are means ± SD (*n* ≥ 30 independent seedlings). The individual images in (E) were digitally extracted for comparison. For (C), (D) and (F), asterisks indicate significant differences compared with mock at *P < 0.05 and **P < 0.01 (Student's *t*-test).

Auxin is well-known to play a key role in plant root growth and development (Olatunji et al., 2017; Brady, 2019; Meier et al., 2020; Yu et al., 2022). Therefore, we asked whether the ABA-inhibited root elongation in rice also requires the function of auxin. To address this question, we used a *DR5-GUS* rice line, which was developed to map auxin distribution in plant tissues (Zhang et al., 2022). Exogenous ABA treatment enhanced GUS activity in the meristematic zone (MZ), elongation zone (EZ) and differentiation zone (DZ) (Figure 2, A and B). Correspondingly, the expression of auxin biosynthesis genes and IAA content were increased after ABA treatment (Figure 2, C and D), indicating that ABA induces the accumulation of auxin in roots by activating the expression of auxin biosynthesis genes.

**Figure 3:**
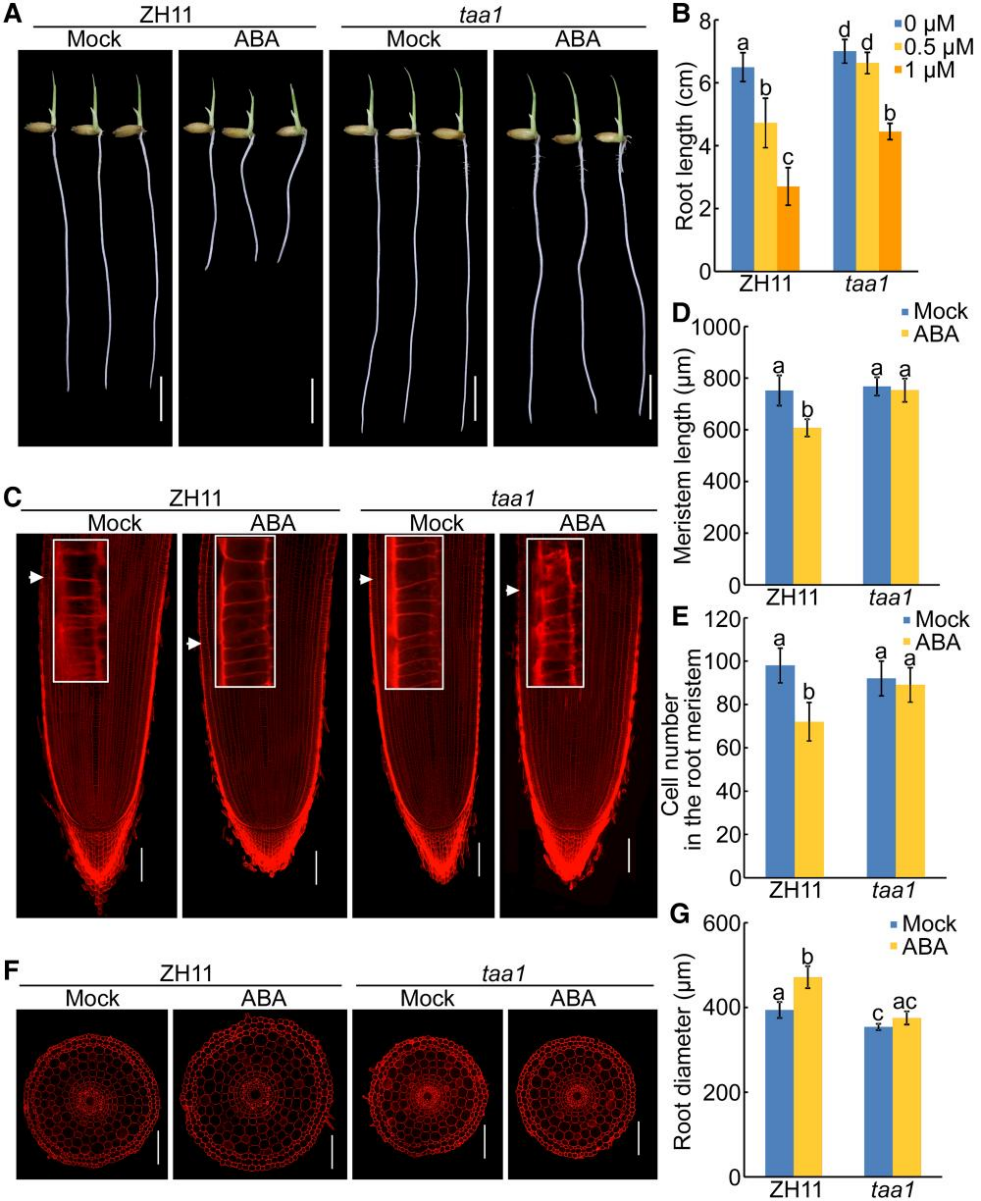
Mutation in *TAA1* weakens ABA response in roots. A, Phenotypes of the primary roots of 4-d-old Zhonghua 11 (ZH11, a wild-type strain) and *taa1* seedlings with or without 0.5 μM ABA treatment. Bar = 1 cm. The individual images were digitally extracted for comparison. B, Primary root length of 4-d-old ZH11 and *taa1* seedlings treated with various concentrations of ABA. The data are shown as mean ± SD, *n* ≥ 30 independent seedlings. C, Representative propidium iodide staining of longitudinal sections of root tips of 4-d-old ZH11 and *taa1* seedlings with or without 0.5 μM ABA treatment. White arrows indicate the proximal end of the root meristem. White rectangle insets are an enlargement (three times magnification) of the regions at the proximal end of the root meristem. Bars = 100 μm. (D and E) Length (D) and cortical cell number (E) of the root meristem zones of the corresponding seedlings indicated in panel (C). Data are means ± SD (*n* ≥ 10 independent seedlings). F, Representative propidium iodide staining of radial sections of root elongation zone of 4-d-old ZH11 and *taa1* seedlings with or without 0.5 μM ABA treatment. Bars = 100 μm. G, Root diameter of the corresponding seedlings indicated in panel (F). Data are means ± SD (*n* ≥ 10 independent seedlings). For (B), (D), (E) and (G), different letters indicate significant differences (*P* < 0.05, one-way ANOVA with Tukey's test).

Next, we treated the seedlings with ABA in the presence of yucasin (an inhibitor of YUC activity) or L-Kyn (a potent inhibitor of TAA1/TAR activity). Our observation showed that ABA-inhibited root elongation in the wild-type seedlings was rescued by yucasin or L-Kyn (Figure 2, E and F), suggesting that intact auxin biosynthesis is required for ABA-inhibited root elongation. To confirm this further, we examined the root phenotype of rice auxin biosynthetic mutants *taa1* (Zhang et al., 2018) and *rein7-1* (Qin et al., 2017) under ABA treatment. Both of them exhibited reduced sensitivity to ABA (Figure 3, A and B and Supplemental Figure S4, A and B). Analysis of longitudinal and transversal sections of root showed that ABA treatment resulted in a decrease in root meristem size (∼18%–20%) and meristem zone cell number (∼24%–26%), and an increase in root diameter (∼17%–35%), whereas this tendency was weakened in the *taa1* and *rein7-1* roots (Figure 3, C–G and Supplemental Figure S4, C–G). These results indicate that ABA inhibits root elongation and promotes root swelling dependent on local auxin biosynthesis.

**Figure 4:**
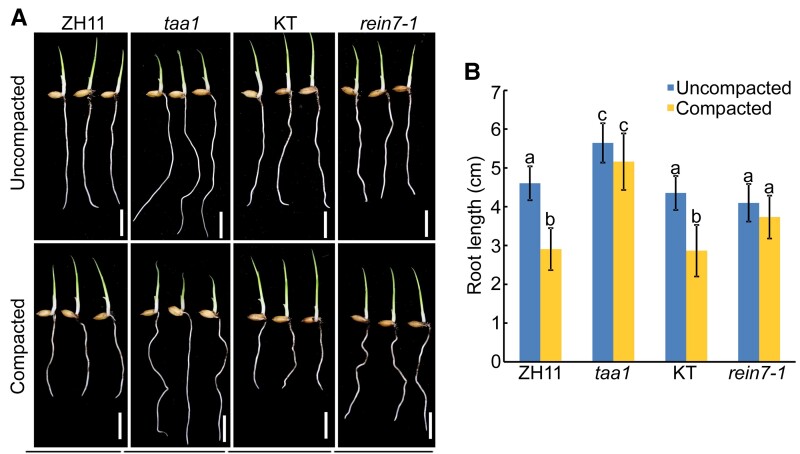
Auxin-deficient mutants display better root penetration ability than wild type in compacted soil. A, Phenotypes of the primary roots of 4-d-old Zhonghua 11 (ZH11, a wild-type strain), *taa1*, Kitaake (KT, a wild-type strain) and *rein7-1* grown in uncompacted and compacted soil. Bar = 1 cm. B, Primary root length of 4-d-old ZH11, *taa1*, KT and *rein7-1* grown in uncompacted and compacted soil. The data are shown as mean ± SD, *n* ≥ 30 independent seedlings. Different letters indicate significant differences (*P* < 0.05, one-way ANOVA with Tukey's test).

The results that ABA mediates root growth in response to soil compaction and *taa1* and *rein7-1* roots exhibit reduced sensitivity to ABA inspired us to investigate whether auxin is involved in ABA-mediated root growth in response to soil compaction. We first investigated the impact of soil compaction on *taa1* and *rein7-1* root growth. Our results showed that both *taa1* and *rein7-1* roots did not exhibit a significant decrease in root length when grown under compacted soil conditions (Figure 4, A and B), suggesting that auxin is critical for triggering root growth responses upon soil compaction. Further detections showed that the expression of auxin biosynthesis genes and IAA content was increased in wild-type roots grown in compacted soil, but this tendency was weakened in *mhz5* roots (Supplemental Figure S5, A and B). These results suggested that auxin is required for ABA-mediated root growth under soil compaction conditions.

### OsbZIP46 positively regulates ABA response in roots

The bZIP transcription factors are major targets of SnRK2 protein kinases in ABA core signaling and play important regulatory roles in activating ABA-dependent gene expression in plants (Tang et al., 2012; Zong et al., 2016; Chang et al., 2017). Since OsbZIP46 is a key regulator in ABA signaling and drought resistance (Tang et al., 2012; Ma et al., 2019), we proposed that OsbZIP46 may be involved in ABA-activated auxin biosynthesis genes expression. To confirm this possibility, we generated loss-of-function mutants of *OsbZIP46* (*osbzip46*) via CRISPR–Cas9, as confirmed by sequencing the target genes. The *osbzip46-1* mutant contained 1-bp insertion in the coding region, triggering a frame shift in the open reading frame, respectively, resulting in early termination. The *osbzip46-2* and *osbzip46-3* knockout lines contained 1-bp and 4-bp deletions in the coding regions of the target genes, leading to a frame shift in the open reading frame and the generation of a premature stop codon (Supplemental Figure S6, A and B). We also generated overexpression (OE) lines containing the *OsbZIP46* coding region under the control of the *CaMV35S* promoter, and the increased expression of the target genes was confirmed by RT-qPCR (Supplemental Figure S6C).

**Figure 5:**
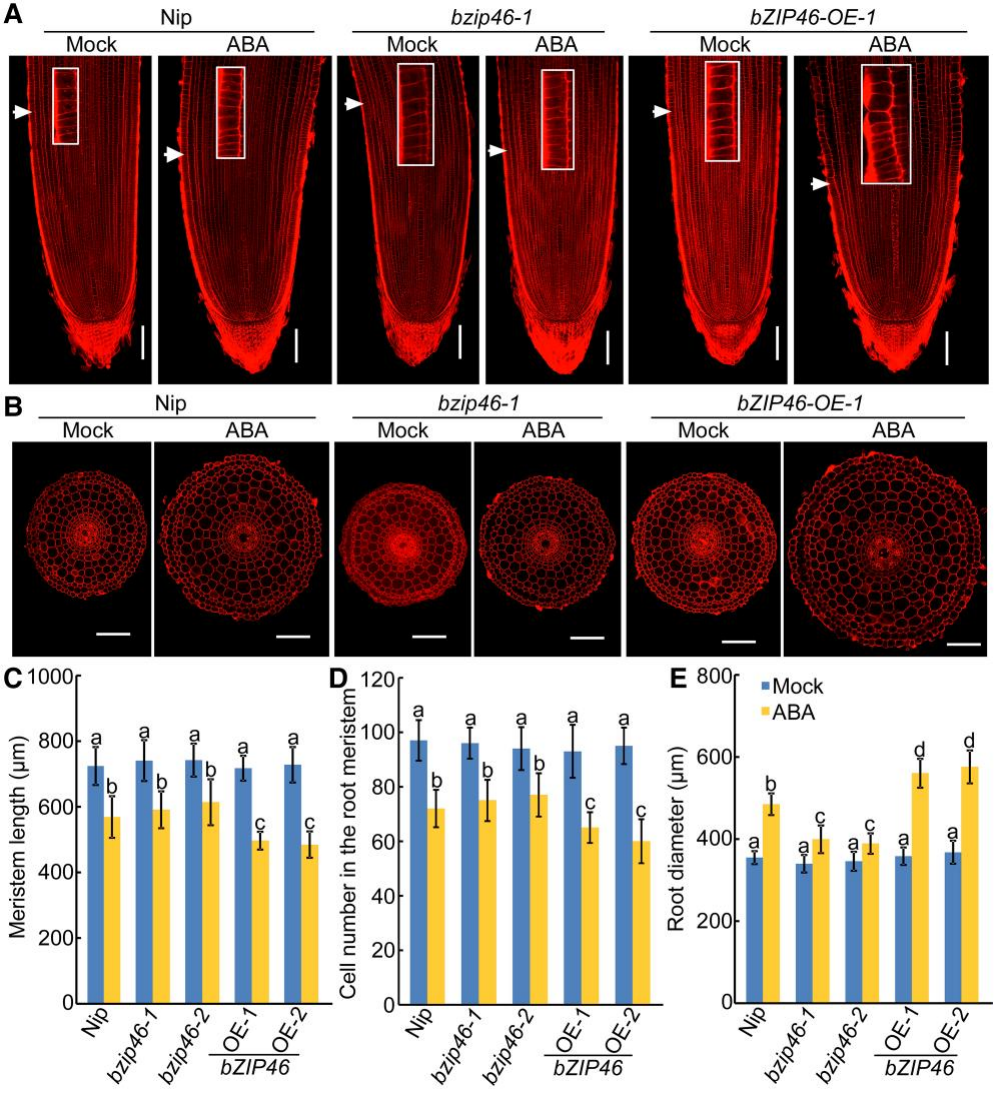
OsbZIP46 positively regulates ABA-modulated root elongation and root swelling. (A and B) Representative propidium iodide staining of longitudinal sections of root tips (A) and radial sections of root elongation zone (B) of 4-d-old Nip, *OsbZIP46* knockout and overexpression seedlings with or without 0.5 μM ABA treatment. White arrows indicate the proximal end of the root meristem. White rectangle insets are an enlargement (three times magnification) of the regions at the proximal end of the root meristem. Bars = 100 μm. (C–E) Length (C) and cortical cell number (D) of the root meristem zones and root diameter (E) of the corresponding seedlings indicated in panels (A) and (B). Data are means ± SD (*n* ≥ 10 independent seedlings). Different letters indicate significant differences (*P* < 0.05, one-way ANOVA with Tukey's test).

Subsequently, we checked the ABA sensitivity of transgenic plants. In the absence of ABA, the root length of *osbzip46* mutants and overexpression lines was similar to that of the wild type (Supplemental Figure S7, A–C). After ABA treatment, the *osbzip46* mutants showed reduced sensitivity to ABA, whereas the overexpression lines showed much shorter roots compared with the wild type (Supplemental Figure S7, A–C). Longitudinal and transversal root tip sections revealed that ABA treatment significantly reduced the meristem size and meristem zone cell number, and increased the root diameter of all seedlings, whereas the *osbzip46* mutants showed a lower increase in root diameter (∼12%–17%) and the *OsbZIP46* overexpression lines showed a higher decrease in meristem size (∼30%–35%) and meristem zone cell number (∼30%–37%), and increase in root diameter (∼56%), as compared with wild-type roots (∼21% decrease in meristem size, ∼26% decrease meristem zone cell number, and 36% increase in root diameter, respectively) (Figure 5, A–E). These results indicate that OsbZIP46 positively regulates ABA response in roots by reducing cell division in root meristem and increasing root diameter in rice.

Previous studies have shown that the native OsbZIP46 had no transactivation activity and deletion of the D domain of OsbZIP46 resulted in constitutive transactivation activity (Tang et al., 2012). Therefore, we further checked the ABA sensitivity of *OsbZIP46-CA1* overexpression seedlings, which were produced by overexpressing a constitutively active form of OsbZIP46 with a deletion of domain D (Tang et al., 2012). The *OsbZIP46-CA1* overexpression seedling roots are significantly shorter than the wild-type roots under normal conditions (Supplemental Figure S8, A–C). Upon ABA treatment, the *OsbZIP46-CA1* overexpression seedling roots showed increased ABA sensitivity (Supplemental Figure S8, A–C), indicating that OsbZIP46 is a positive regulator of ABA signaling depending on its activation.

Next, we examined the root phenotype of *osbzip46* mutants, *OsbZIP46* overexpression seedlings, and *OsbZIP46-CA1* overexpression seedlings in response to soil compaction. Soil compaction significantly reduced the root length in wild-type seedlings, with a milder phenotype in *osbzip46* mutants and a more pronounced phenotype in *OsbZIP46* and *OsbZIP46-CA1* overexpression seedlings (Supplemental Figure S9, A–D), indicating that the OsbZIP46-mediated pathway is partially required for the regulation of the soil compaction-induced inhibition of root growth.

### OsbZIP46 activates the expression of *OsYUC8/REIN7* through direct binding to its promoter

**Figure 6:**
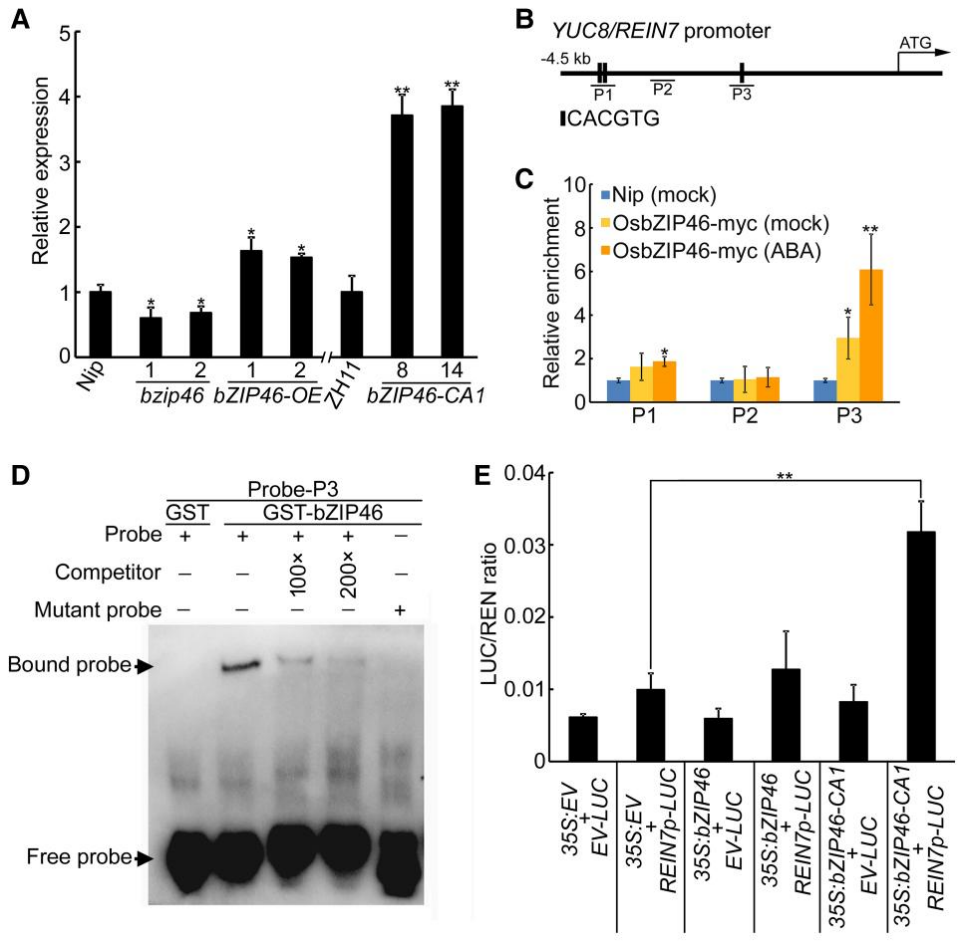
OsbZIP46 directly binds to the promoter of *YUC8/REIN7* to activate its expression. A, Expression of *YUC8/REIN7* in the roots of 4-d-old wild type, *OsbZIP46* knockout and overexpression seedlings, and *OsbZIP46-CA1* overexpression seedlings. The data are shown as mean ± SD; *n* = 3 biological replicates. Asterisks indicate significant differences compared with Nip or ZH11 at *P < 0.05 and **P < 0.01 (Student's *t*-test). B, Schematic diagram of G-box (5′-CACGTG-3′) in the *YUC8/REIN7* promoter. Black boxes indicate the positions of the G-box. P1–P3 are *YUC8/REIN7* promoter fragments. C, The enrichments of *YUC8/REIN7* promoter analyzed by ChIP-qPCR using *35S:OsbZIP46-myc* transgenic rice plants. Wild-type plants were used as a negative control. The data are shown as mean ± SD, *n* = 3 biological replicates. Asterisks indicate significant differences compared with Nip values at * *P* < 0.05 and ** *P* < 0.01 (Student's *t*-test). D, EMSA of OsbZIP46 binding to *YUC8/REIN7* promoter region containing the G-box. Normal G-box and the mutated form (5′-AAAAAA-3′) are shown. Competition was done by adding an excess of unlabeled probe (Competitor), and for specificity with labeled mutant probe. Three biological replicates were performed, with similar results. E, Dual-LUC assay results from transient transformation of rice mesophyll protoplasts with constructs constitutively expressing *OsbZIP46* or *OsbZIP46-CA1* and the LUC reporter gene under control of the intact *YUC8/REIN7* promoter. The data are shown as mean ± SD, *n* = 3 biological replicates. “EV” represents empty vector. The asterisks indicate significant differences by Student's *t*-test between two samples (**P < 0.01).

The data that OsbZIP46 positively regulates ABA response in roots and ABA induces the expression of auxin biosynthesis genes and the accumulation of auxin in roots prompt us to examine whether OsbZIP46 regulates the expression of auxin biosynthesis genes in roots. To verify this, we detected the expression of *TAA* and *YUC* genes in *osbzip46* mutants, *OsbZIP46* and *OsbZIP46-CA1* overexpression seedling roots. Our results showed that the expression of *YUC2*, *YUC3*, *YUC8/REIN7*, and *TAA1* was regulated by OsbZIP46 (Figure 6A and Supplemental Figure S10, A and B), implying that *YUC2*, *YUC3*, *YUC8/REIN7*, and *TAA1* might be potential targets of OsbZIP46.

Previous studies have shown that bZIP superfamily transcription factors preferentially bind a G-box upstream of target genes to regulate downstream gene expression (Zong et al., 2016). We then analyzed the promoter sequence of *YUC2*, *YUC3*, *YUC8/REIN7*, and *TAA1* and identified three G-box (5′-CACGTG-3′) in *YUC8/REIN7* promoter (Figure 6B). Hence, we performed a chromatin immunoprecipitation (ChIP) assay using transgenic plants harboring myc-tagged OsbZIP46 (OsbZIP46-myc). As shown in Figure 6C, OsbZIP46 was significantly enriched in the P3 fragment of the *YUC8/REIN7* promoter, while there was no significant enrichment in the other fragments (Figure 6C). Subsequently, we performed the electrophoresis mobility shift assay (EMSA) using the recombinant OsbZIP46 protein. The results showed that OsbZIP46 bound to the P3 fragment of the *YUC8/REIN7* promoter, but it did not bind to the DNA probe with mutated G-box (5′-AAAAAA-3′) (Figure 6D). Non-biotin-labeled DNA fragment effectively competed with the binding (Figure 6D). These results indicate that OsbZIP46 directly binds to the *YUC8/REIN7* promoter in vivo and in vitro.

To determine whether OsbZIP46 activates the expression of *YUC8/REIN7*, we performed a transient expression assay in which we fused the 4,500-bp promoter sequence upstream of the ATG codon of *YUC8/REIN7* to the *LUCIFERASE* (*LUC*) reporter gene and cotransfected rice protoplasts with the effector plasmid harboring *35S:bZIP46* or *35S:bZIP46CA1*. Compared with the control vector, the full-length OsbZIP46 protein has no obvious effect on LUC activity driven by the *YUC8/REIN7* promoter, whereas OsbZIP46-CA1 drastically elevated the level of LUC activity (Figure 6E), this is consistent with previous reports that the native OsbZIP46 protein cannot be able to activate the expression of downstream genes (Tang et al., 2012). Taken together, these results indicate that OsbZIP46 directly binds to the promoter of *YUC8/REIN7* to activate its expression.

### *YUC8/REIN7* functions downstream of *OsbZIP46* in ABA-controlled root growth

**Figure 7:**
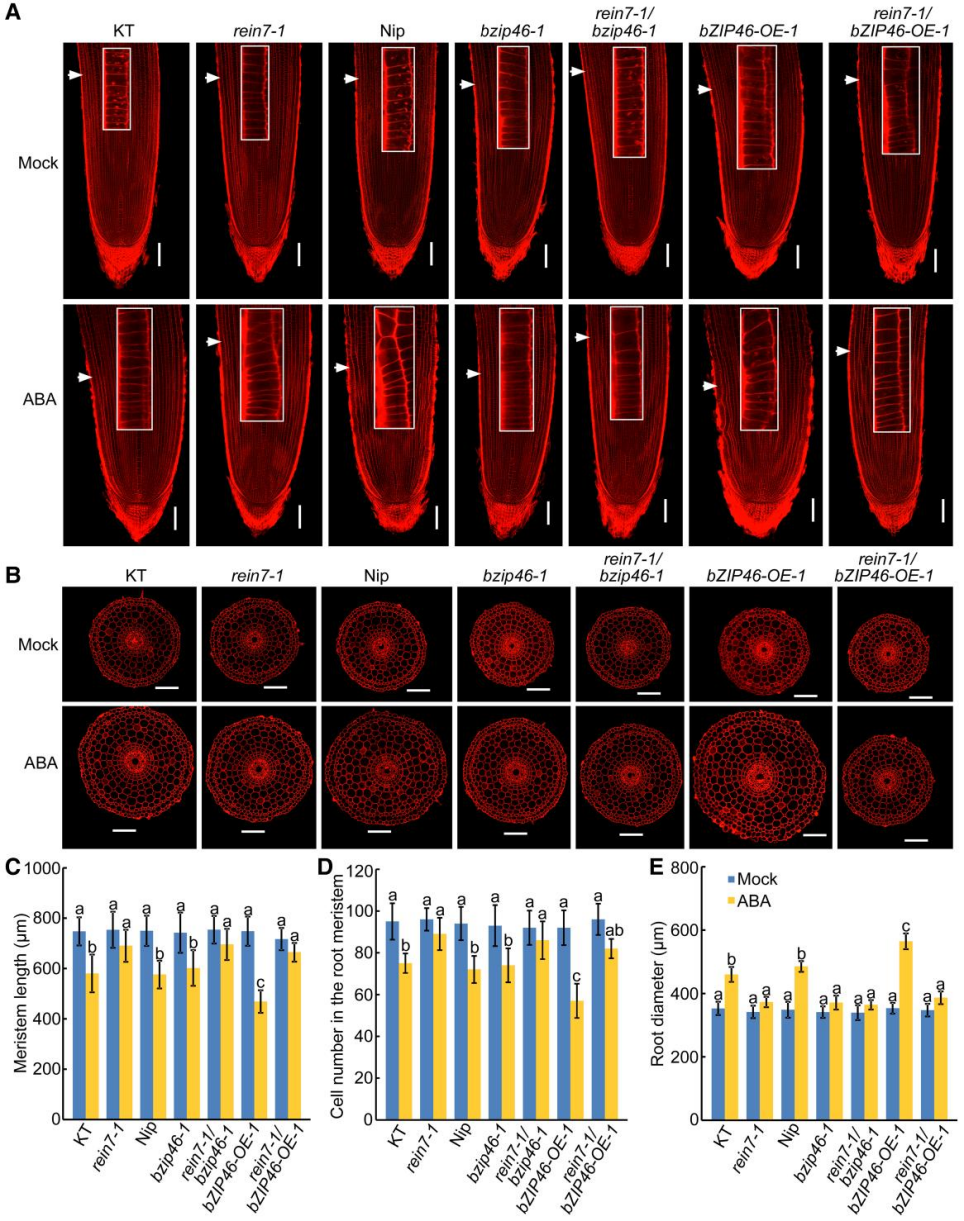
*YUC8/REIN7* acts downstream of *OsbZIP46* to regulate ABA-modulated root elongation and root swelling. (A and B) Representative propidium iodide staining of longitudinal sections of root tips (A) and radial sections of root elongation zone (B) of 4-d-old Nip, and combinations of *YUC8/REIN7* and *OsbZIP46* knockout and overexpression seedlings with or without 0.5 μM ABA treatment. White arrows indicate the proximal end of the root meristem. White rectangle insets are an enlargement (three times magnification) of the regions at the proximal end of the root meristem. Bars = 100 μm. (C–E) Length (C) and cortical cell number (D) of the root meristem zones and root diameter (E) of the corresponding seedlings indicated in panels (A) and (B). Data are means ± SD (*n* ≥ 10 independent seedlings). Different letters indicate significant differences (*P* < 0.05, one-way ANOVA with Tukey's test).

To explore the genetic relationship between *OsbZIP46* and *YUC8/REIN7*, we firstly generated double mutant by crossing *rein7-1* with *bzip46-1*. Without ABA treatment, the root length of the *rein7-1 bzip46-1* double mutant was similar to that of the *rein7-1* mutant and *bzip46-1* mutant (Supplemental Figure S11, A–C). With ABA treatment, the roots of the *rein7-1*, *bzip46-1*, and *rein7-1 bzip46-1* double mutant all displayed reduced sensitivity to exogenous ABA (Supplemental Figure S11, A–C). Correspondingly, the *rein7-1 bzip46-1* double mutant showed a lower decrease in meristem size (∼8%), meristem zone cell number (∼6%), and increase in root diameter (∼7%) under ABA treatment, as compared with wild type (∼23% decrease in meristem size, ∼21% decrease in meristem zone cell number, and ∼30%–39% increase in root diameter, respectively), similar to that of *rein7-1* mutant (Figure 7, A–E). These results suggest that *OsbZIP46* and *YUC8/REIN7* most likely act within the same pathway for ABA-modulated root growth.

To further examine the genetic relationship between *OsbZIP46* and *YUC8/REIN7*, we then analyzed the ABA response of the *rein7-1 bZIP46-OE-1* plants that were obtained by crossing *rein7-1* with *bZIP46-OE-1*. The roots of *rein7-1* and *rein7-1 bZIP46-OE-1* homozygous plants were less inhibited than the wild type and *bZIP46-OE-1* seedlings when exposed to ABA (Supplemental Figure S11, A–C). Anatomical analysis of root apices revealed that *rein7-1* and *rein7-1 bZIP46-OE-1* plants had bigger root meristem size, higher meristem zone cell number, and smaller root diameter than wild type and *bZIP46-OE-1* seedlings under ABA treatment (Figure 7, A–E). These data suggest that *YUC8/REIN7* acts downstream of *OsbZIP46* and *YUC8/REIN7*-mediated pathway are partially required by OsbZIP46 signaling for the regulation of the ABA-modulated root growth.

## Discussion

Roots anchor plants and take up water and nutrient from the soil; therefore, root development strongly affects plant growth and productivity (de Dorlodot et al., 2007; Uga et al., 2013). As the belowground organ of the plant, roots encounter varying environmental conditions and respond by altering their growth, and plant hormones play an important role in this process (Meier et al., 2020; Lv et al., 2021b; Pandey et al., 2021; Taylor et al., 2021). Soil compaction is a serious global problem causing inadequate rooting and poor yield in crops around the world (Hamza and Anderson, 2005; Correa et al., 2019). Soil compaction can occur in both top and subsoil. In agricultural soils, the main factors responsible for compaction are excessive traffic, the use of farm equipment that exceeds the bearing capacity of soil, and tillage at unsuitable soil water contents, in particular wet soils (Correa et al., 2019). Rice is a semi-aquatic plant that grows in a water-saturating environment for most of its life cycle; this means that rice is more susceptible to soil compaction. Thus, dissecting the molecular mechanism of soil compaction restricting root growth will help us improve the performance of crops under specific agronomical conditions. In the present study, we report that auxin acts downstream of ABA to inhibit root elongation and promote root swelling in response to soil compaction, and the ABA signaling transcription factor OsbZIP46 functions as a crosstalk node between ABA and auxin by directly activating the expression of auxin biosynthesis gene *YUC8/REIN7*. Thus, our results unravel a molecular mechanism that bridges ABA signaling and auxin biosynthesis in primary root development, providing avenues for breeders to select crops resilient to soil compaction.

The roots continuously grow and develop through balancing cell division and cell differentiation within the root apical meristem, thus root apical meristem is an origin for longitudinal root growth (Jiang and Feldman, 2005; Perilli et al., 2012; Petricka et al., 2012). In the present study, we found that ABA treatment reduced root meristem size, meristem zone cell number, and maturation zone cell length, thereby restricting root elongation, indicating that ABA plays an important role in maintaining root meristem activity and cell elongation. In addition to reducing root elongation, we also found that ABA treatment promoted root tip swelling, which was mainly caused by the expansion of cortical cells. This dual effect of ABA on root growth depends on auxin. Increasing studies show that the site of action of different hormones can mainly be accounted for by single cell types: the epidermis for auxin and cortical cells for ABA, and root growth is coordinated by different types of cells (Swarup et al., 2005; Ubeda-Tomas et al., 2009; Dietrich et al., 2017; Vaseva et al., 2018). Here, we show that ABA employs auxin to control longitudinal and horizontal root growth, demonstrating that OsbZIP46 acts as a mediator to translate ABA signal into auxin biosynthesis to control root growth. Thus, our results step up the understanding of the coordination of ABA and auxin in root elongation and root swelling.

ABA is a key phytohormone that plays pivotal roles in root growth (Harris, 2015; Sun et al., 2018; Zhang et al., 2021). In *Arabidopsis*, low concentrations of ABA stimulate but high concentrations inhibit primary root elongation, and both effects depend on auxin (Li et al., 2017). However, the molecular mechanism of how ABA signal is translated into auxin is largely unknown. In this study, we showed that ABA transcriptionally regulates the expression of *YUC8/REIN7*, an auxin biosynthesis gene (Fujino et al., 2008; Qin et al., 2017), thereby inhibiting primary root elongation and promoting primary root swelling in rice. This conclusion is supported by the following evidence: (1) ABA treatment induces the expression of auxin biosynthesis genes and auxin accumulation in roots; (2) disruption auxin biosynthesis by L-Kyn or yucasin rescued ABA-inhibited primary root elongation; (3) primary roots of auxin biosynthesis deficient mutants exhibit reduced sensitivity to ABA; (4) ABA signaling transcription factor OsbZIP46 directly binds to the *YUC8/REIN*7 promoter to activate its transcription; and (5) *YUC8/REIN7* acts downstream of *OsbZIP46* to modulate primary root elongation and swelling in response to ABA. Thus, the *YUC8/REIN7*-mediated auxin biosynthesis pathway is required for ABA-controlled root growth.

The bZIP transcription factor family is one of the most diverse families in vascular plants. Rice genome contains 89 putative bZIPs, which can be classified into 11 groups based on their amino acid sequence similarity and DNA-binding specificity (Nijhawan et al., 2008). OsbZIP46 is one member of the third subfamily of bZIP transcription factors in rice. It has high sequence similarity to ABSCISIC ACID-INSENSITIVE5 (OsABI5) and OsbZIP23, two transcription factors play important roles in ABA and stress response in rice (Zou et al., 2007, 2008; Zong et al., 2016), suggesting that they may have functional redundancy in regulating ABA and stress response. In the present study, we showed that mutation in *OsbZIP46* does not affect root growth under normal conditions, and has a very limited effect on root growth under ABA treatment and compacted soil conditions, suggesting that the OsbZIP46-mediated pathway is partially required for the regulation of the ABA- and soil compaction-induced inhibition of root growth, and other bZIPs beside bZIP46 may also participate in this process.

Soil compaction represents a major agronomic challenge, inhibiting root growth and resource capture, causing substantial yield losses (Lipiec et al., 2012; Correa et al., 2019). In this study, we show that soil compaction inhibits root elongation and promotes root swelling. Deep rooting is favorable for the acquisition of water and nitrogen from the subsoil (Lynch, 2013), whereas swelling root is advantageous for water and nutrients flux and root branching (Li et al., 2015a). Thus, the dual role of soil compaction on root growth is conducive for achieving optimal growth and higher grain production under adverse conditions in rice. Previous studies have shown that compacted soil restricts diffusion of ethylene, thereby causing the accumulation of ethylene in root tissues and triggering hormone responses that restrict growth (Pandey et al., 2021; Huang et al., 2022). Accumulating evidence indicates that auxin and ABA function downstream of ethylene to inhibit root elongation (Ma et al., 2014; Yin et al., 2015; Qin et al., 2017). In the present study, we evidence that ABA and auxin are also involved in the regulation of root growth in response to soil compaction. Disruption of ABA or auxin biosynthesis leads to higher root penetration ability when grown in compacted soil. Thus, our results provide a pathway for breeders to improve plant performance in crops under edaphic stress.

**Figure 8:**
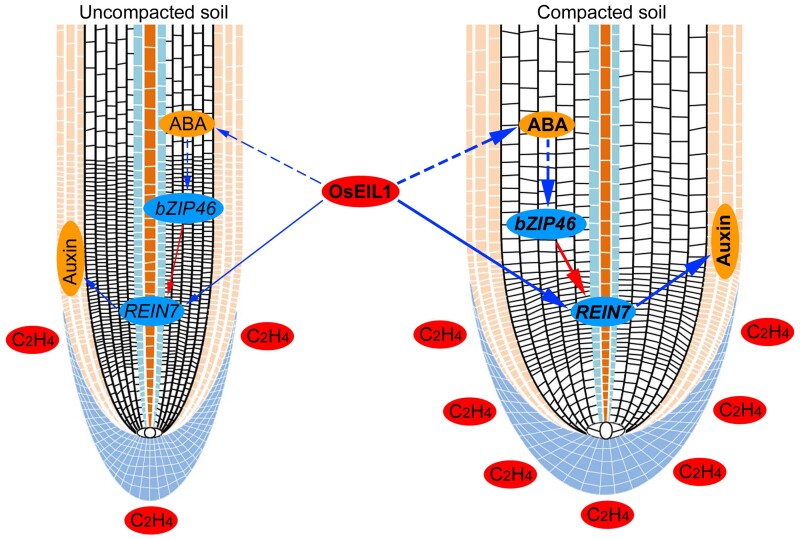
The proposed model of root responses in uncompacted soil and compacted soil. Compacted soil condition promotes higher ethylene response due to restricted diffusion of ethylene, leading to the accumulation of OsEIL1 in the roots. OsEIL1 directly activates *YUC8* to promote auxin biosynthesis in epidermal cells, thus inhibiting root elongation. In parallel, OsEIL1 activates ABA biosynthesis in cortical cells, causing radial expansion of root cortical cells and root swelling. Increased ABA mediating OsbZIP46 activates the expression of *YUC8* to promoting auxin biosynthesis, which further inhibits epidermal cell elongation and root elongation, ultimately resulting in short and swollen roots. The solid lines indicate direct interactions, the dashed lines indicate indirect interactions, the blue lines indicate regulatory relationships reported in previous studies, the red lines indicate regulatory relationships reported in this study, and the thickness of the lines indicates the strength of regulation.

OsEIL1 encodes a critical transcription factor in the ethylene transduction pathway, and both ethylene and soil compaction promotes the accumulation of OsEIL1 protein in roots (Yang et al., 2015; Pandey et al., 2021). Previous studies have demonstrated that OsEIL1 directly binds to the promoter of *YUC8/REIN7* to activate its expression, leading to accumulation of auxin in roots and inhibition of root growth (Qin et al., 2017). In this study, we evidenced that ABA mediating OsbZIP46 activates the expression of *YUC8/REIN7* to modulate root growth. Thus, YUC8/REIN7 acts as a node for ethylene and ABA to regulate root growth in response to soil compaction. Studies in *Arabidopsis* show that auxin mainly acts on epidermis and ABA on cortical to control root growth (Vaseva et al., 2018). Increasing studies in rice report that ABA and auxin function downstream of ethylene to inhibit root elongation. Based on our present data and previous reports (Ma et al., 2014; Yin et al., 2015; Qin et al., 2017; Pandey et al., 2021; Huang et al., 2022), we propose a modulatory model that ethylene orchestrates induction of both ABA and auxin to regulate root growth in response to soil compaction (Figure 8). Compacted soil restricts the diffusion of ethylene, leading to the accumulation of OsEIL1 in roots, which directly up-regulates auxin biosynthesis through *YUC8/REIN7*. Higher auxin levels in epidermal cells inhibit epidermal cell elongation, resulting in a reduction in meristem size and inhibition of root elongation. In parallel, OsEIL1 also activates ABA biosynthesis in cortical cells, causing radial expansion of root cortical cells and root swelling. Higher ABA mediating OsbZIP46 activates the expression of *YUC8/REIN7* to promote auxin biosynthesis, which further inhibits epidermal cell elongation and root elongation, ultimately resulting in short and swollen root (Figure 8). Our results shed mechanistic insights into how roots adapt to soil compaction via ethylene-mediated up-regulation of auxin and ABA biosynthesis, which further enriches the regulatory network of root development on the basis of previous studies (Huang et al., 2022).

## Materials and methods

### Plant materials and growth conditions

Rice (*O. sativa*) *rein7-1*, *taa1*, *mhz5*, *DR5-GUS* transgenic line, and *OsbZIP46-CA1* overexpression lines were described previously (Tang et al., 2012; Yin et al., 2015; Qin et al., 2017; Zhang et al., 2018). *osbzip46* allelic mutants in the Nip background were generated using CRISPR/Cas9 (Xu et al., 2017), in which the target regions 5′-CATCGACCCGAAATCCTTCT-3′ of *OsbZIP46* was introduced into the pHUN4c12 vector backbone, and the recombinant vector was transformed into *Agrobacterium tumefaciens* EHA105-pSOUP for rice transformation. To generate the overexpression transgenic plants, the coding sequence of *OsbZIP46* was amplified by PCR and cloned in-frame with a myc-tag into pCAMBIA1307 under the control of the *CaMV35S* promoter (Wang et al., 2020). The resulting plasmids were introduced into Nip via *Agrobacterium*-mediated transformation. The primers used are listed in Supplemental Table S1.

To analyze root growth, the seeds were submerged in water at 37°C for 48 h. The germinated seeds were sown in a stainless steel sieve plate in a container of Yoshida's culture solution (Cui et al., 2015). The plants were housed in a growth chamber under a 14-h light (30°C)/10-h dark (25°C) photoperiod, with a light intensity of ∼150 μmol m^−2^ s^−1^ (white light) and 60% relative humidity. ABA, yucasin, and L-Kyn treatments were performed as previously described (Qin et al., 2017). Briefly, the germinated seeds were incubated in hydroponic solution containing various concentrations of ABA or 5 μM yucasin/L-Kyn for 4 d. ABA was dissolved in ethanol, yucasin and L-Kyn were in DMSO. The controls were conducted with treatments containing equivalent volumes of ethanol or DMSO. At the end of the period, the roots were scanned and their length was measured from digitized images using Image J software.

### Soil compaction experimental

The soil compaction experimental was performed as previously described with minima modification (Wang et al., 2019). Briefly, nutrient soil was passed through a sieve with a 2-mm mesh size and then mixed with vermiculite (v/v = 2:1). Subsequently, the soil was lightly sprayed with sterilized water until the moisture content of the damp soil reached 80% (80 ml sterilized water per 100 g soil), mixed thoroughly and stored in dark for three to four days to room temperature to equilibrate. In no compaction treatment group, the glass cylinders (20 cm × 6 cm) were filled with soil until the total height of soil column was 10 cm. In the compaction treatment group, double volume of uncompacted soil was compressed to heights of 10 cm. Germinated rice seeds were placed on the soil surface and covered with a 1-cm top layer of loose soil in both uncompacted and compacted soil column. Seedlings were grown for 4 d in a growth chamber under a 14-h light (30°C)/10-h dark (25°C) photoperiod, with a light intensity of ∼150 μmol m^−2^ s^−1^ (white light) and 60% relative humidity. Root phenotype was observed by flushing the soil with tap water, and root length was analyzed via ImageJ software.

### Reverse transcription quantitative PCR (Rt-qPCR)

Total RNA was extracted from 4-d-old seedlings root using an Ultrapure RNA Kit (CWBIO) according to manufacturer's instructions. Approximately 2 μg total RNA was reverse transcribed to cDNA with HiScript II Q RT SuperMix (Vazyme) according to manufacturer's instructions. RT-qPCR was performed as previously described (Zhang et al., 2012), using the rice *Actin1* gene as an internal standard to normalize gene expression. The RT-qPCR primers are listed in Supplemental Table S1.

### β-Glucuronidase (GUS) staining

For GUS staining, roots were collected from 4-d-old *DR5-GUS* transgenic plants, incubated in GUS staining buffer (50 mM sodium phosphate, pH 7.0; 10 mM EDTA; 0.5 mM K_3_[Fe (CN)_6_]; 0.5 mM K_4_[Fe (CN)_6_]; 0.1% [v/v] Triton X-100) containing 1 mM 5-bromo-4-chloro-3-indolyl-β-D-glucuronic acid at 37°C for 6 h. After the samples were rinsed with 70% ethanol until the tissue cleared, they were photographed. To produce transverse section of roots, root segments were embedded in 3% agar. Transverse sections (30 μm) of root were produced using a vibratome (Leica VT 1000S). The images of rice root autofluorescence were taken under a microscope (Axiocam 506 color).

### Vibrotome and confocal imaging

Rice tissue sections were generated as previously described (Truernit et al., 2008). Root apices (∼5 mm) of 4-d-old rice seedlings were harvested and fixed in 50% (v/v) methanol, 10% (v/v) glacial acetic acid at 4°C for at least 12 h, rinsed with distilled water, and incubated in 1% (v/v) periodic acid at room temperature for 40 min. Root tissue was again rinsed with water and incubated in Schiff reagent with PI (100 mM sodium metabisulphite and 0.15 N HCl; PI freshly added to a final concentration of 100 μg/ml) until plants were visibly stained.

To obtain cross-sectional images, the root segments were embedded in 3% (w/v) agar and transverse sections (40 μm) were cut with a vibratome (Leica VT 1000 S). Samples were transferred onto microscope slides and covered with a chloral hydrate solution (4 g chloral hydrate, 1 ml glycerol, and 2 ml water), and subsequently imaged with a confocal laser-scanning microscope (ZEISS LSM980) using an excitation wavelength of 543 nm. Root meristem size and cell number were determined as described previously (Li et al., 2015b). Briefly, root meristem size was defined by measuring the length from the quiescent center to the first elongated epidermal cell. Cell number in the root meristem was determined by counting cortical cells from the quiescent center to the first expanding cortical cells in the fourth cortical layer of the root meristem.

### Chromatin immunoprecipitation (ChIP)-qPCR assay

The ChIP-qPCR assay was performed as previously described (Saleh et al., 2008). 4-d-old 35S-myc-*OsbZIP46* transgenic seedling roots and anti-myc antibodies (Abmart) were used for ChIP experiments. Briefly, the roots were ground into a fine powder with liquid nitrogen and resuspended in nuclei isolation buffer. The nucleus were then collected by centrifugation and resuspended with nuclei lysis buffer. The resuspended chromatin was sonicated to a size of 200–500 bp subsequently. myc-*OsbZIP46* was precipitated from input DNA with anti-myc antibodies or without any antibodies. Protein A agarose beads (Millipore) were added into the incubation mixture for additional 2 h at 4°C. The immune complexes were eluted from the washed protein A beads. The DNA was purified with phenol/chloroform (v/v = 1:1) and precipitated. The purified DNA and input DNA were used as templates. The enrichments of DNA fragments were analyzed by qPCR with specific primers listed in Supplemental Table S1.

### Electrophoretic mobility shift (EMSA) assay

To produce the OsbZIP46 protein, the coding sequence of *OsbZIP46* was fused with GST-coding sequence and inserted into the pGEX-6p-1 vector. The resulting vector was expressed in *Escherichia coli* strain BL21(DE3) and the recombinant protein was purified using a *ProteinIso* GST resin (Transgen) according to the manufacturer's instructions. Oligonucleotide probes of the *YUC8/REIN7* promoter were synthesized and labeled with biotin at their 3′ end (Sangon Biotech). EMSA was performed using a LightShift Chemiluminescent EMSA Kit (Thermo Fisher). Briefly, reaction solutions were incubated for 20 min at room temperature. The protein-probe mixture was separated on a 5% (v/v) polyacrylamide native gel and transferred to a nylon membrane (GE). Following crosslinking under UV light, the DNA on the membrane was detected using a Chemiluminescent Nucleic Acid Detection Module (Thermo Fisher).

### Luciferase transient expression assay

To quantitatively analyses normalized luciferase (LUC/REN) activity, rice protoplasts were prepared and transfected with the corresponding constructs via polyethylene glycol-mediated transfected as previously described (Bart et al., 2006). Firefly luciferase (LUC) and Renilla luciferase (REN) activities were measured with a dual-LUC reporting assay kit (Promega). LUC activity was normalized to REN activity and the relative LUC/REN ratios were calculated.

### IAA content measurement

IAA was quantified as previously described (Qin et al., 2017). Briefly, roots of 4-d-old seedlings treated with or without 1 μM ABA for 24 h were collected, quickly frozen in liquid nitrogen and ground into a fine powder, and 50 mg powder was extracted with 1 ml methanol/H_2_O/formic acid (15:4:1, v/v/v) containing 10 ng/ml d5-IAA. The combined extracts were evaporated to dryness under nitrogen gas stream, reconstituted in 100 μl 80% methanol (v/v), and filtered through 0.22 μm filters. Quantification was performed in an AB 6500 + QTRAP® LC–MS/MS system (Applied Biosystems, United States) with stable, isotope-labeled auxin as the standard (OlChemIm, Czech Specials) according to a method described previously (Wang et al., 2017).

### Accession numbers

Sequence data from this article can be found in the GenBank database under the following accession numbers: *OsbZIP46*, Os06g10880; *YUC1*, Os01g45760; *YUC2*, Os05g45240; *YUC3*, Os01g53200; *YUC4*, Os01g12490; *YUC5*, Os12g32750; *YUC6*, Os07g25540; *YUC7*, Os04g03980; *YUC8*, Os03g06654; *YUC9*, Os01g16714; *YUC10*, Os01g16750; *YUC11*, Os12g08780; *YUC12*, Os02g17230; *YUC13*, Os11g10140; *YUC14*, Os11g10170; *TAA1*, Os01g07500; *TAR1*, Os05g07720; *OsActin1*, Os03g50885.

## Supplementary Material

kiac586_Supplementary_Data

## Supplemental data

The following materials are available in the online version of this article.

**Supplemental Figure S1.** Soil compaction inhibits cell elongation in maturation zone.

**Supplemental Figure S2.** ABA inhibits root elongation and promotes root swelling.

**Supplemental Figure S3.** ABA inhibits cell elongation in maturation zone.

**Supplemental Figure S4.** Y*U*C8*/REIN7*-mediated auxin biosynthesis is required for ABA-modulated root elongation and root swelling.

**Supplemental Figure S5.** Soil compaction induces higher expression of auxin biosynthesis genes and auxin accumulation is weakened in *mhz5* mutant roots.

**Supplemental Figure S6.** Identification of *OsbZIP46* mutants and overexpression lines.

**Supplemental Figure S7.** O*s*bZIP46 positively regulates ABA response in roots.

**Supplemental Figure S8.** Overexpression of the constitutively active (CA) form of *OsbZIP46* enhanced ABA response in roots.

**Supplemental Figure S9.** OsbZIP46-mediated pathway is partially required for soil compaction-inhibited primary root growth.

**Supplemental Figure S10.** OsbZIP46 positively regulates the expression of auxin biosynthesis genes in roots.

**Supplemental Figure S11.** Y*U*C8*/REIN7* acts downstream of *OsbZIP46* to regulate ABA response in roots.

**Supplemental Table S1.** Primers used in this study.

## Author responsibility

The author responsible for distribution of materials integral to the findings presented in this article in accordance with the policy described in the Instructions for Authors (https://academic.oup.com/plphys/pages/General-Instructions) is Hua Qin (qinhua@caas.cn).
