# Supplementary material for: Abscisic acid promotes auxin biosynthesis to inhibit primary root elongation in rice
Source: Plant Physiol. 2022 Dec 19;191(3):1953–67. doi: 10.1093/plphys/kiac586 (PMC10022642; doi:10.1093/plphys/kiac586)
Supplement: kiac586_Supplementary_Data [file kiac586_supplementary_data.pdf]

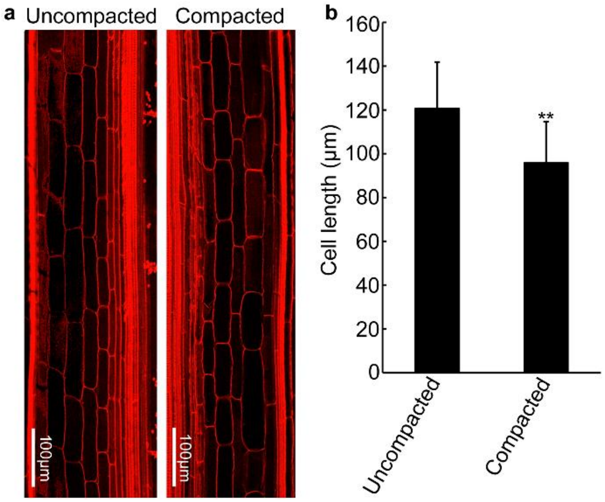

Supplemental Figure S1. Soil compaction inhibits cell elongation in maturation zone. (a) Representative propidium iodide staining of longitudinal sections of root maturation zones of 4-day-old WT seedlings grown in uncompacted or compacted soil. Bars = 100 μm. (b) Cell length of the root maturation zone of the corresponding seedlings indicated in panel (a). Data are means ± SD (n ≥ 10 independent seedlings). Asterisks indicate significant differences compared with uncompacted at $^{**}P < 0.01$ (Student's *t*-test).

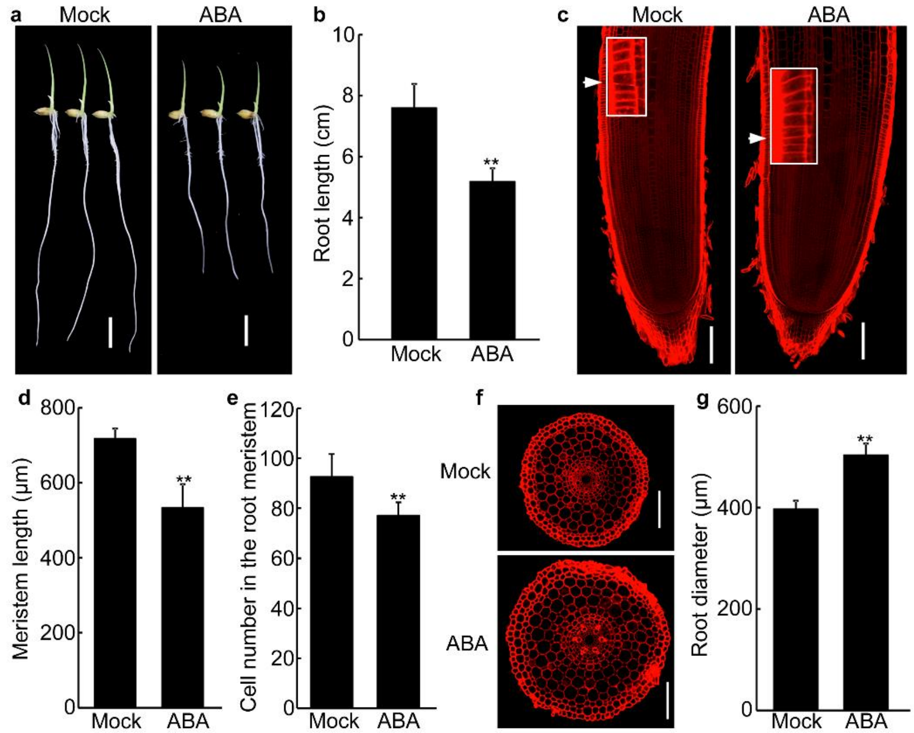

Supplemental Figure S2. ABA inhibits root elongation and promotes root swelling. (a and b) Morphology (a) and primary root length (b) of 4-day-old wild-type (WT) seedlings with or without 0.5 µM ABA treatment. Bars = 1 cm. Data are means ± SD (n ≥ 30 independent seedlings). (c) Representative propidium iodide staining of longitudinal sections of root tips of 4-day-old WT seedlings with or without 0.5 µM ABA treatment. White arrows indicate the proximal end of the root meristem. White rectangle insets are an enlargement (3 times magnification) of the regions at the proximal end of the root meristem. Bars = 100 µm. (d and e) Length (d) and cortical cell number (e) of the root meristem zones of the corresponding seedlings indicated in panel (c). Data are means ± SD (n ≥ 10 independent seedlings). (f) Representative propidium iodide staining of radial sections of root elongation zone of 4-day-old WT seedlings with or without 0.5 µM ABA treatment. Bars = 100 µm. (g) Root diameter of the corresponding seedlings indicated in panel (f). Data are means ± SD (n ≥ 10 independent seedlings). Asterisks in (b), (d), (e) and (g) indicate significant differences compared with mock at $^{**}P < 0.01$ (Student's *t*-test).

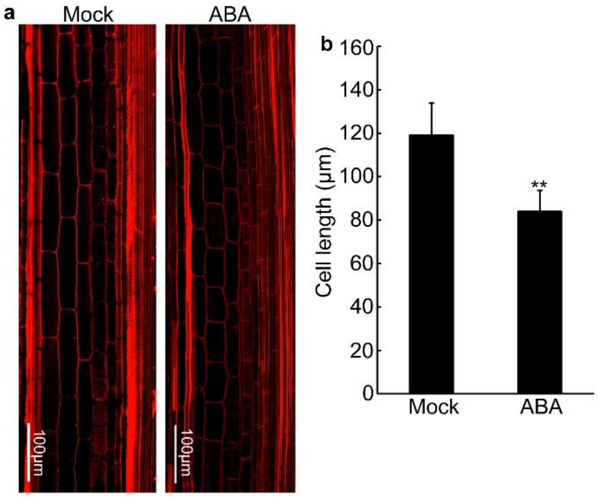

Supplemental Figure S3. ABA inhibits cell elongation in maturation zone. (a) Representative propidium iodide staining of longitudinal sections of root maturation zones of 4-day-old WT seedlings with or without 0.5 µM ABA treatment. Bars = 100 µm. (b) Cell length of the root maturation zone of the corresponding seedlings indicated in panel (a). Data are means ± SD ($n \geq 10$ independent seedlings). Asterisks indicate significant differences compared with mock at $^{**}P < 0.01$ (Student's *t*-test).

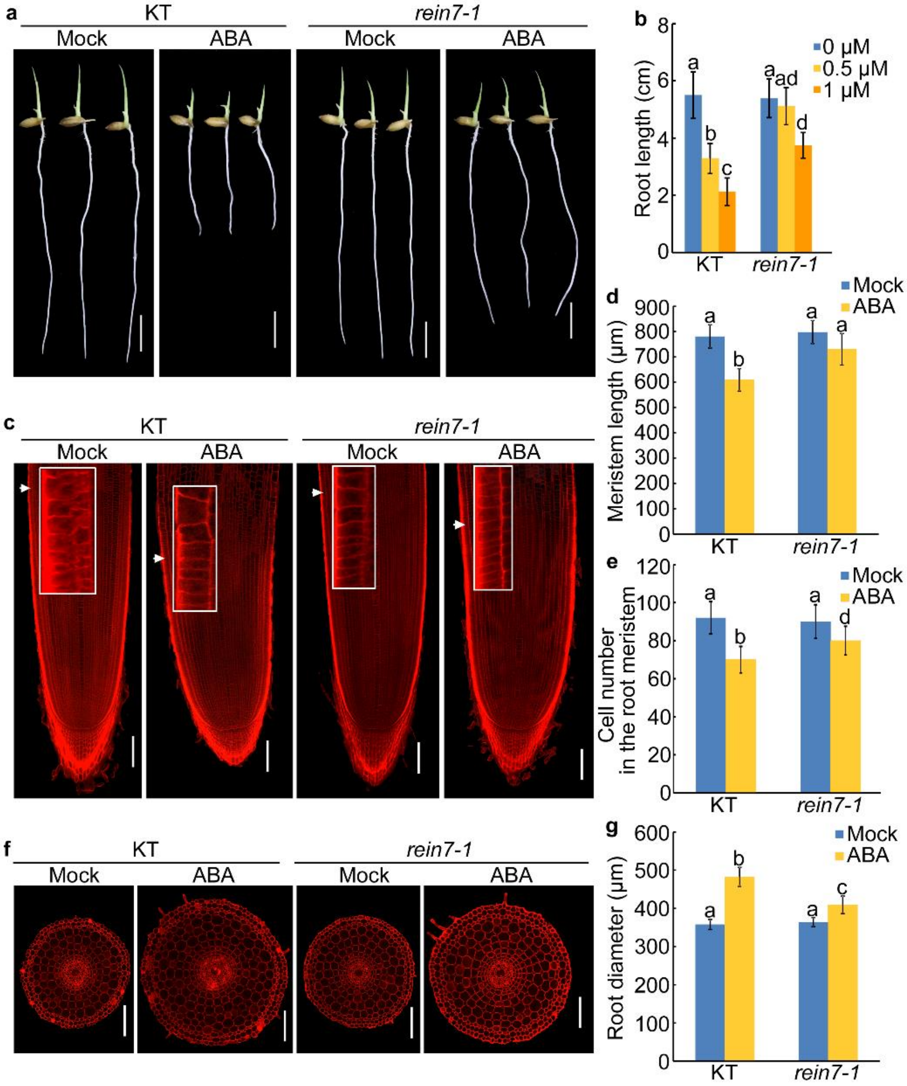

Supplemental Figure S4. *YUC8/REIN7*-mediated auxin biosynthesis is required for ABA-modulated root elongation and root swelling. (a) Phenotypes of the primary roots of 4-day-old Kitaake (KT, a wild-type strain) and *rein7-1* seedlings with or without 0.5 μM ABA treatment. Bar = 1 cm. (b) Primary root length of 4-day-old KT and *rein7-1* seedlings treated with various concentrations of ABA. The data are shown as mean ± SD, n ≥ 30 independent seedlings. (c) Representative propidium iodide staining of longitudinal sections of root tips of 4-day-old KT and *rein7-1* seedlings with or without 0.5 μM ABA treatment. White arrows indicate the proximal end of the root meristem. White rectangle insets are an enlargement (3 times magnification) of the regions at the proximal end of the root meristem. Bars = 100 μm. (d and e) Length (d) and cortical cell number (e) of the root meristem zones of the corresponding seedlings indicated in panel (c). Data are means ± SD (n ≥ 10 independent seedlings). (f) Representative propidium iodide staining of radial sections of root elongation zone of 4-day-old KT and *rein7-1* seedlings with or without 0.5 μM ABA treatment. Bars = 100 μm. (g) Root diameter of the corresponding seedlings indicated in panel (f). Data are means ± SD (n ≥ 10 independent seedlings). For (b), (d), (e) and (g), different letters indicate significant differences ($P < 0.05$, one-way ANOVA with Tukey's test).

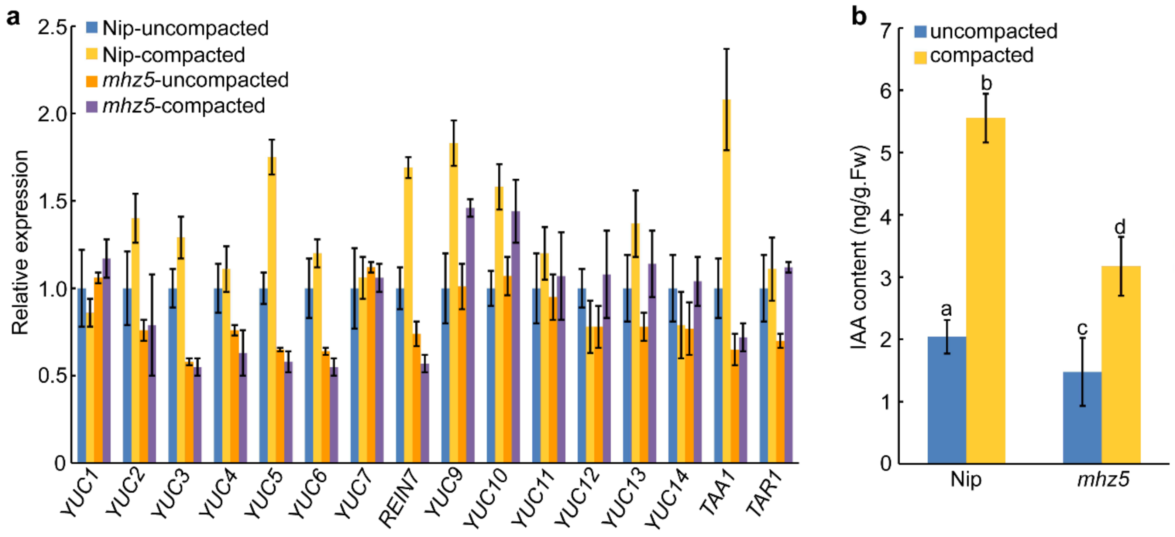

Supplemental Figure S5. Soil compaction induces higher expression of auxin biosynthesis genes and auxin accumulation is weakened in *mhz5* mutant roots. (a) Expression of *YUCs*, *TAA1* and *TAR1* in the roots of 4-d-old Nip and *mhz5* seedlings grown in uncompacted or compacted soil. The data are shown as mean ± SD; n = 3 biological replicates. (b) Indole-3-acetic acid (IAA) contents in 4-d-old Nip and *mhz5* seedlings grown in uncompacted or compacted soil. The data are shown as mean ± SD; n = 3 biological replicates. Different letters indicate significant differences ($P < 0.05$, one-way ANOVA with Tukey's test).

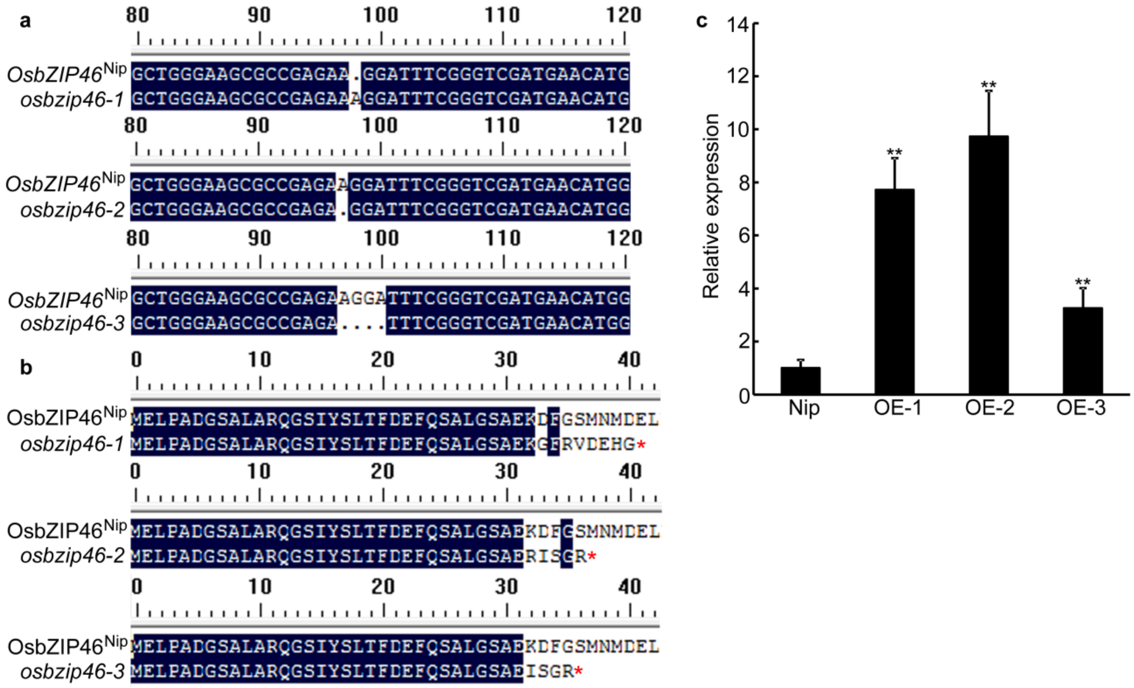

Supplemental Figure S6. Identification of *OsbZIP46* mutants and overexpression lines. (a) DNA sequences of *OsbZIP46* in *osbzip46* allelic mutants (*osbzip46-1*, *osbzip46-2*, and *osbzip46-3*) generated by CRISPR/Cas9. (b) Amino acid sequences of *OsbZIP46* in *osbzip46* allelic mutants. (c) Expression analysis of *OsbZIP46* in overexpression lines. Values indicate ± SD (n = 3). ** indicates significant differences by Student's *t*-test compared to Nip at $P < 0.01$.

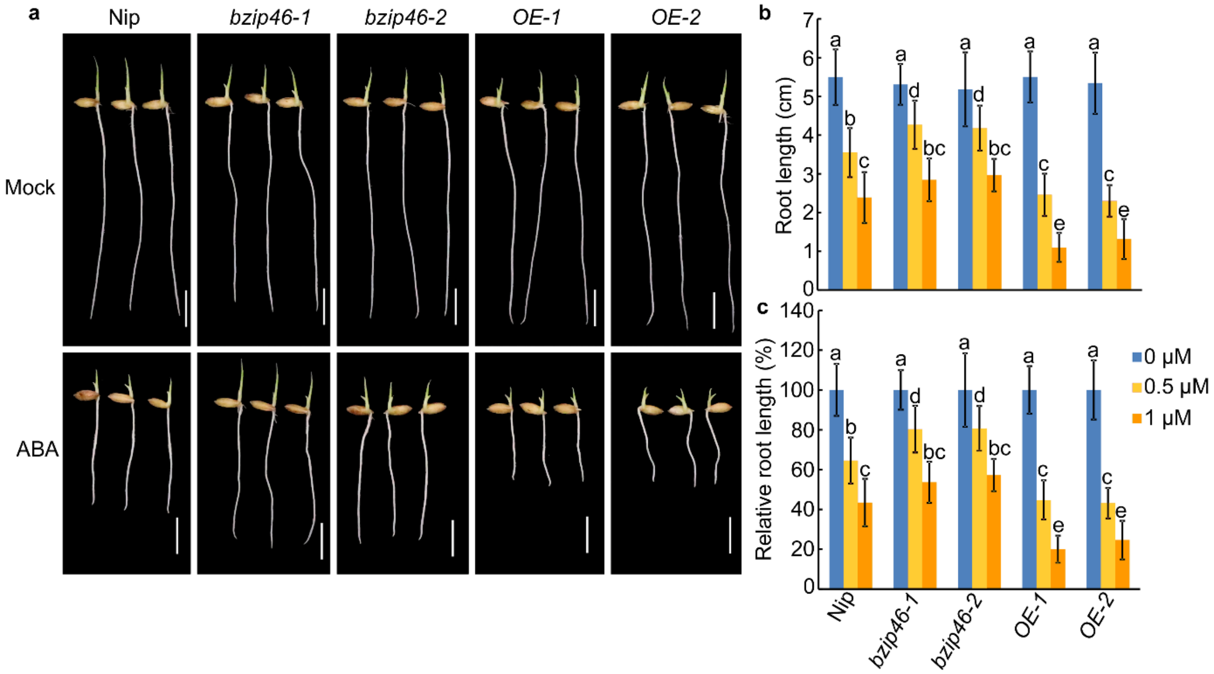

Supplemental Figure S7. *OsbZIP46* positively regulates ABA response in roots. (a) Phenotypes of the primary roots of 4-day-old Nip, *OsbZIP46* knockout and overexpression seedlings with or without 0.5 μM ABA treatment. Bar = 1 cm. (b and c) Primary root length (b) and relative root length (c) of 4-day-old Nip, *OsbZIP46* knockout and overexpression seedlings treated with various concentrations of ABA. The data are shown as mean ± SD, n ≥ 30 independent seedlings. Different letters indicate significant differences ($P < 0.05$, one-way ANOVA with Tukey's test).

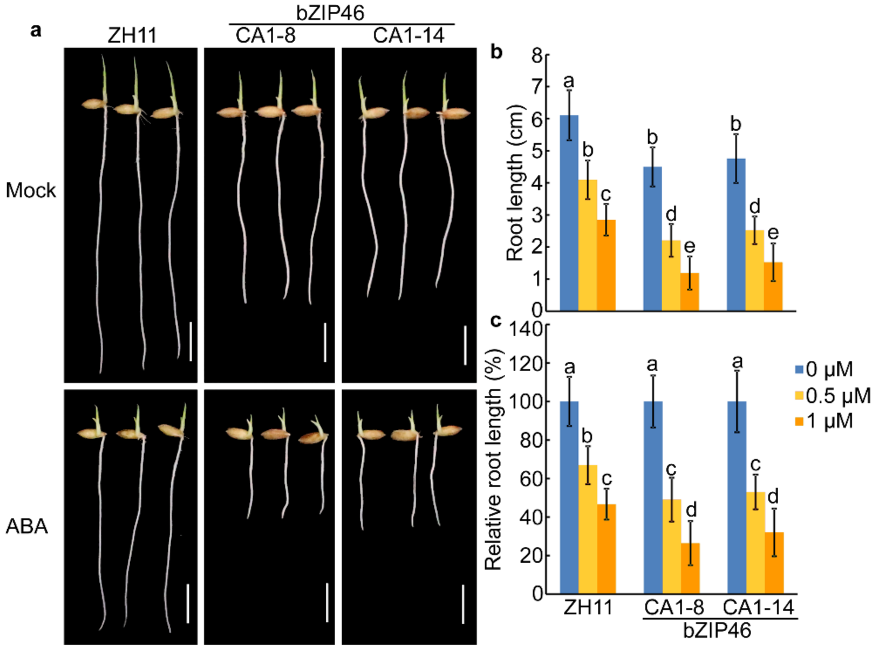

Supplemental Figure S8. Overexpression of the constitutively active (CA) form of *OsbZIP46* enhanced ABA response in roots. (a) Phenotypes of the primary roots of 4-day-old Zhonghua 11 (ZH11, a wild-type strain) and *OsbZIP46-CA1* overexpression seedlings with or without 0.5 μM ABA treatment. Bar = 1 cm. (b and c) Primary root length (b) and relative root length (c) of 4-day-old ZH11 and *OsbZIP46-CA1* overexpression seedlings treated with various concentrations of ABA. The data are shown as mean ± SD, n ≥ 30 independent seedlings. Different letters indicate significant differences ($P < 0.05$, one-way ANOVA with Tukey's test).

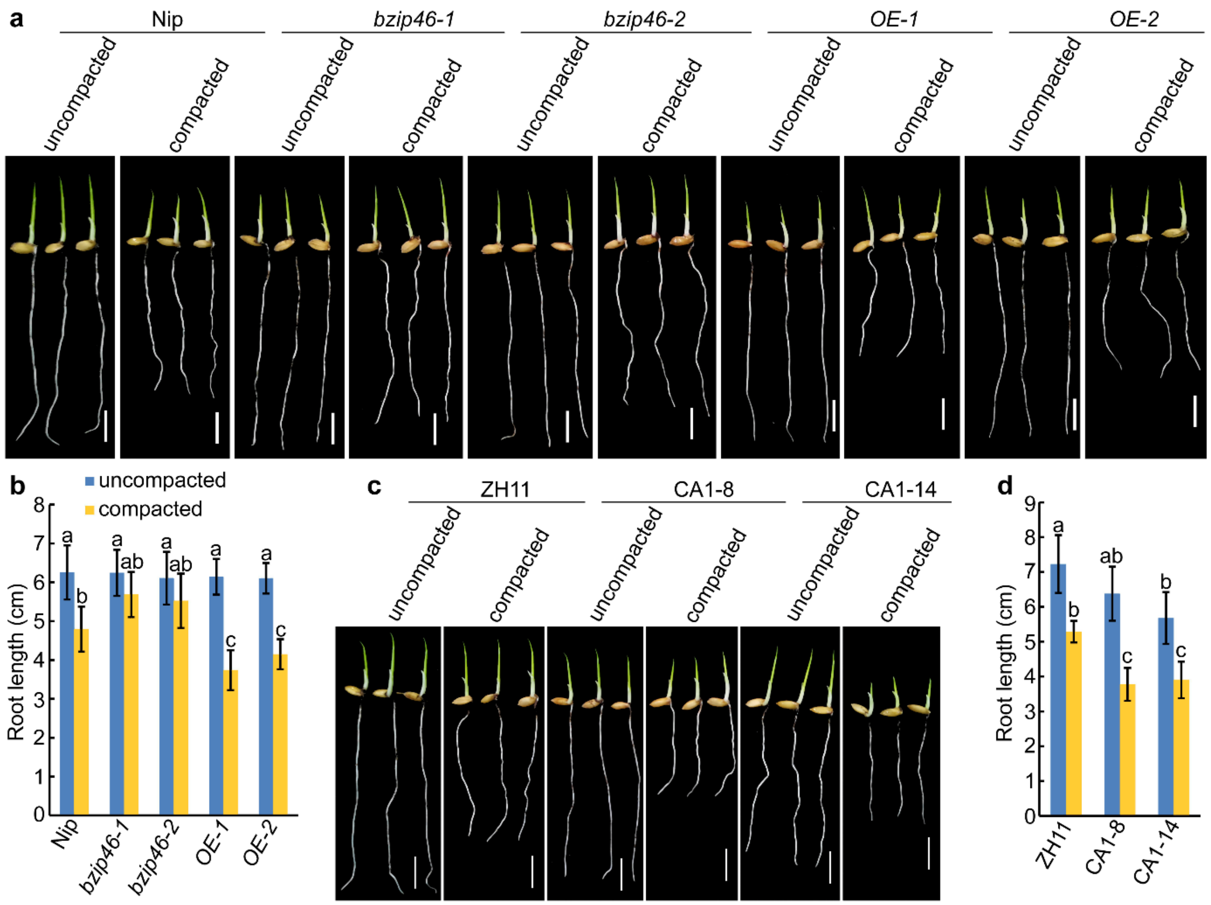

Supplemental Figure S9. OsbZIP46-mediated pathway is partially required for soil compaction-inhibited primary root growth. (a-d) Morphology (a and c) and primary root length (b and d) of 4-day-old Nip, *OsbZIP46* knockout and overexpression seedlings, Zhonghua 11 (ZH11, a wild-type strain) and *OsbZIP46-CA1* overexpression seedlings grown in uncompacted or compacted soil. Bars = 1 cm. Data are means ± SD (n ≥ 30 independent seedlings). Different letters indicate significant differences ($P < 0.05$, one-way ANOVA with Tukey's test).

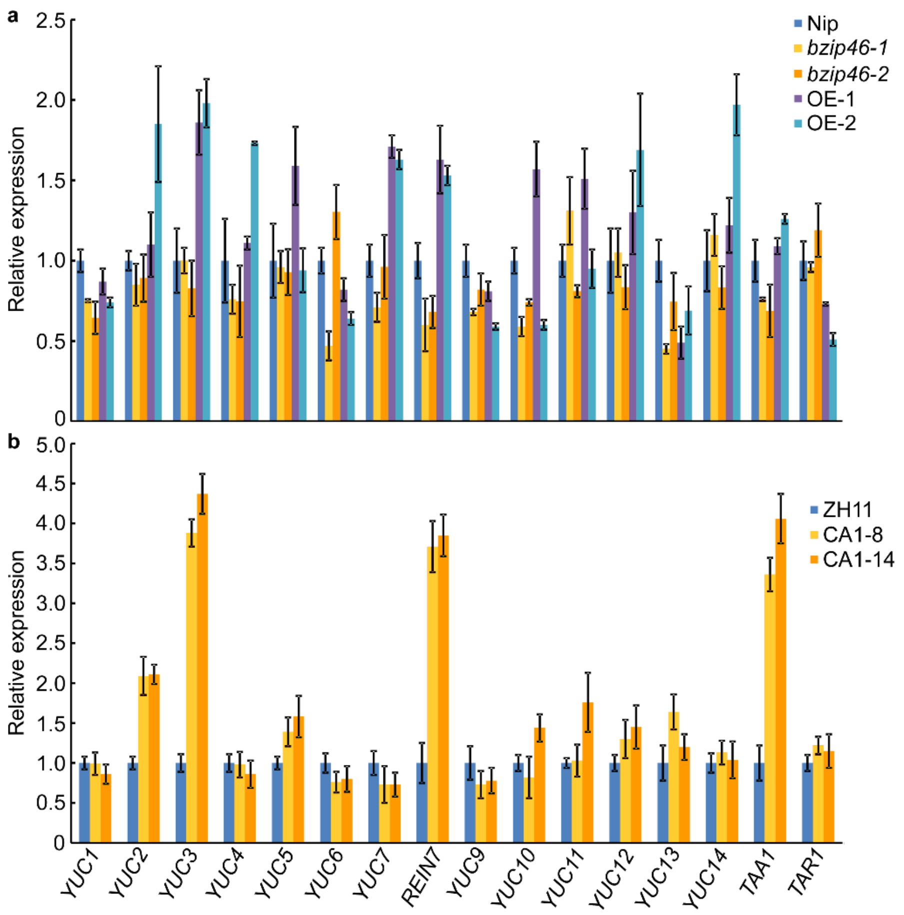

Supplemental Figure S10. OsbZIP46 positively regulates the expression of auxin biosynthesis genes in roots. (a and b) Expression of auxin biosynthesis genes in the roots of 4-day-old Nip, *OsbZIP46* knockout and overexpression seedlings, Zhonghua 11 (ZH11, a wild-type strain), and *OsbZIP46-CA1* overexpression seedlings. The data are shown as mean ± SD, n = 3 biological replicates.

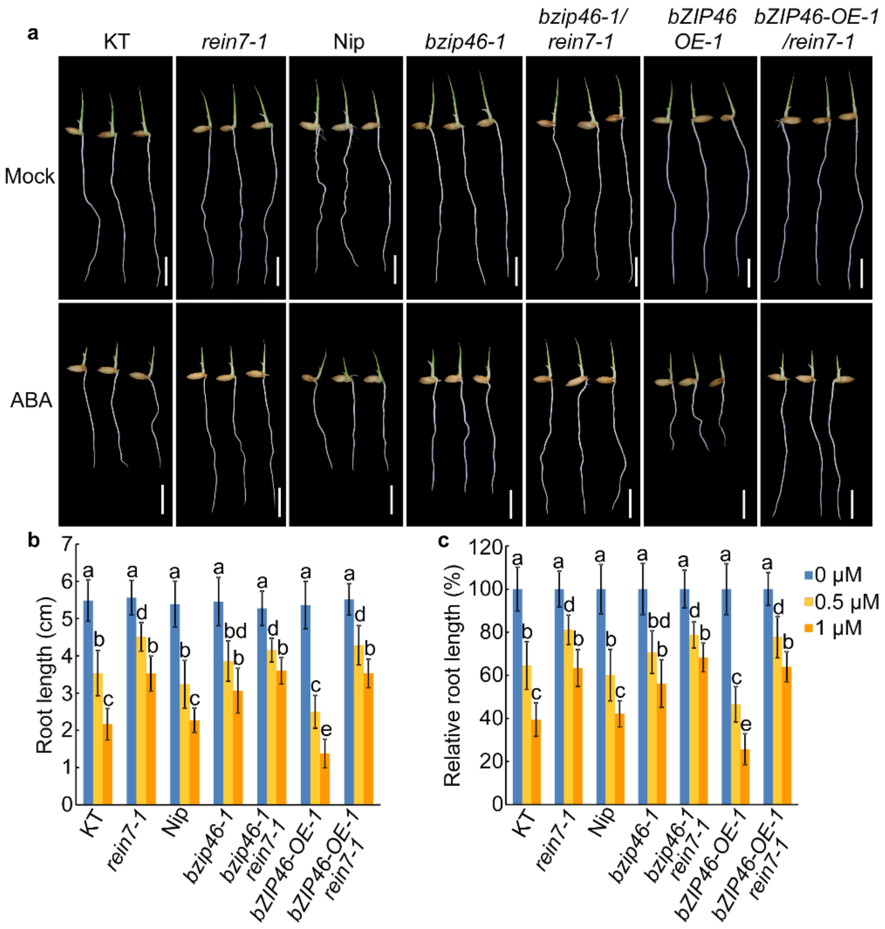

Supplemental Figure S11. *YUC8/REIN7* acts downstream of *OsbZIP46* to regulate ABA response in roots. (a) Phenotypes of the primary roots of 4-day-old Nip, and combinations of *YUC8/REIN7* and *OsbZIP46* knockout and overexpression seedlings with or without 0.5 µM ABA treatment. Bar = 1 cm. (b and c) Primary root length (b) and relative root length (c) of 4-day-old Nip, and combinations of *YUC8/REIN7* and *OsbZIP46* knockout and overexpression seedlings treated with various concentrations of ABA. The data are shown as mean ± SD, n ≥ 30 independent seedlings. Different letters indicate significant differences ($P < 0.05$, one-way ANOVA with Tukey's test).

Supplemental Table S1. Primers used in this study.

**Primer**	**Sequence** (5'-3')
**RT-qPCR analysis**	
OsActin1-F	GACCTTGCTGGGCGTGAT
OsActin1-R	GTCATAGTCCAGGGCGATGT
YUC1-F	TCATCGGACGCCCTCAACGTCGC
YUC1-R	GGCAGAGCAAGATTATCAGTC
YUC2-F	CAACTCTGGCATGGAGGTGT
YUC2-R	GGATCGGCAACCATTTGAGC
YUC3-F	GTGAGAACGGGCTCTACTCGGTCG
YUC3-R	GCTTATGCATGACCGATGAACACG
YUC4-F	CTCGACCTCTGCAACCACAA
YUC4-R	GGAGTACCAGCCAATCCACC
YUC5-F	GAGAAATACGGCCTCCGACG
YUC5-R	CGACCCCATCCTCTGTGAAG
YUC6-F	CCATTCCCAGATGGTTGGAAGG
YUC6-R	CATGTTGCGCCTCAAGATATTTG
YUC7-F	CAACGGATGGAAGGGTGAGT
YUC7-R	CTTCTTGGTTGGCTTCGTCG
YUC8-F	CCAACATCTCCTCGGTGTAG
YUC8-R	GCATCAGACAAGCAACATCC
YUC9-F	CACGATGTACCCGGTCGTG
YUC9-R	TCTCCCCCTTCCAGTGATCC
YUC10-F	TTACCGGAGCACCACCAAG
YUC10-R	GTTGCTGATGTGGCCATTGTA
YUC11-F	ATGCCCAAGAAGGACTTCCC
YUC11-R	GAAGGCCTTGACGTCATTAGCA
YUC12-F	CATGCACCGCATCGTTATGG
YUC12-R	CCGTGCAGTGAGTCTTGCTA
YUC13-F	CCACACATGGCGTATCCTGT
YUC13-R	CAGCCCAACGGTTCTTACCT
YUC14-F	TAGGTTCGAGGGAGAAGCCA
YUC14-R	GTACCGGGCTACGAACAACT
TAA1-F	CGCCCTACTACTCGTCATACC
TAA1-R	GATTGTTCGGGGAGCAGAC
TAR1-F	GCACCATACTACTCCTCGTACCC
TAR1-R	GACGAGCTCGACGTAGGTGT
OsbZIP46-F	AGCAGGTGGAAATGATACAG
OsbZIP46-R	GGTCCAAGTTGCTGAGTGATTC
**To generate the *OsbZIP46* overexpression vectors**	
OsbZIP46-OE-F	ATGTCGACATGGAGTTGCCGGCGGATGG
OsbZIP46-OE-R	ATGGTACCCCATGGACCAGTCAGTGTTC

**CRIPSR/Cas9-mediated gene editing of *OsbZIP46***	
OsbZIP46-cas9-F	GGCACATCGACCCGAAATCCTTCT
OsbZIP46-cas9-R	AAACAGAAGGATTTCGGGTCGATG
**For dual-luciferase assays**	
YUC8p-LUC-F	ATGTCGACCGAGAGAGAGAGGGAGAAACA
YUC8p-LUC-R	ATGGATCCCGATCGATCGATGGCCCAAG
**For ChIP-qPCR**	
YUC8-P1F	CGAGAGAGAGAGGGAGAAACA
YUC8-P1R	TTCGAATCCACTCCAAATCA
YUC8-P2F	ATATAAAATTTAGTACCTCCTG
YUC8-P2R	TGCATATATCCAGATTTAAGC
YUC8-P3F	GCACTATTGAGAATCGATTCA
YUC8-P3R	ACGAATTTAACTCTCAGGAATT
**For EMSA**	
OsbZIP46-GST-F	ATGGATCCATGGAGTTGCCGGCGGATGG
OsbZIP46-GST-R	ACGTCGACCCATGGACCAGTCAGTGTTC
YUC8-Probe	GTTGGAAAGCGTATTTTATTCGCA**ACGTG**CGGTTCTTCCAGTTTCTCGGC
YUC8-Mutant Probe	GTTGGAAAGCGTATTTTATTCGCA**AAAAA**CGGTTCTTCCAGTTTCTCGGC
